# Online LIBS–ML Framework for Dynamic Characterization of Heterogeneous Waste-Derived Gasification Feedstocks

**DOI:** 10.1021/acsomega.6c03040

**Published:** 2026-05-20

**Authors:** Ozan Karadut, Javidan Aliyev, Carlos E. Romero, Zheng Yao, Robert De Saro, Joseph Craparo

**Affiliations:** † Energy Research Center, 1687 Lehigh University, Bethlehem, Pennsylvania 18015, United States; ‡ Department of Mechanical Engineering and Mechanics, 1687 Lehigh University, Bethlehem, Pennsylvania 18015, United States; § Department of Chemical and Biomolecular Engineering, 1687 Lehigh University, Bethlehem, Pennsylvania 18015, United States; ∥ Energy Research Company, 400 Leland Ave, Plainfield, New Jersey 07062, United States

## Abstract

LIBS–ML framework for real time feedstock characterization during continuous conveyor transport Heterogeneous waste derived feedstocks (e.g., waste coal, biomass and blends) introduce rapid variability in heating value and ash chemistry that affect gasifier operation, yet conventional laboratory characterization techniques are too slow to support proactive control. To address this gap, this study reports on an online, in situ, dynamic characterization framework that couple’s laser-induced breakdown spectroscopy (LIBS) with leakage safe machine learning (ML) regression to deliver real time, decision quality predictions of gasifier relevant properties. A controlled sample matrix spanning two different waste coals, two different biomasses, and engineered blends under two particle size conditions were constructed and benchmarked using standardized laboratory analyses for proximate/ultimate properties and ash composition. LIBS spectra were acquired dynamically as material flowed on a conveyor belt, using high energy 1064 nm laser ablation and shot averaging to improve repeatability and precision. Supervised regression models (multi layer perceptron (MLP) /artificial neural network (ANN), random forest (RF), and support vector regression (SVR)) and an optimized weighted ensemble were trained on emission line feature sets using nested cross validation with Bayesian hyperparameter tuning and validated against an independent hold out set. The proposed LIBS–ML workflow achieves near laboratory predictive fidelity across parametric targets (including higher heating value (HHV), ash content, fixed carbon, sulfur, major ash forming oxides, and initial deformation temperature (IDT)), with the weighted ensemble providing a robust default predictor under dynamic measurement conditions. These results demonstrate a practical pathway for real time feedstock characterization that can enable feedforward adjustments and more resilient gasifier operation for variable quality waste derived fuels.

## Graphical abstract

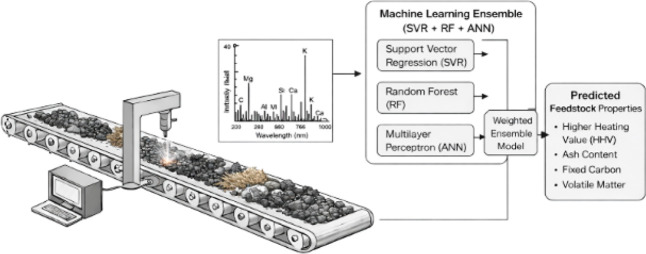

## Introduction

Gasification is a versatile thermochemical process that has gained popularity because of its flexibility in both technology and end products. This technology is receiving a renaissance as a primary pathway for global warming mitigation, hydrogen production as a clean energy carrier, and for allowing for better emissions control by capturing pollutants before combustion.^1^ Gasification converts carbon based raw materials into syngas using gasifying agents such as steam, air, and/or oxygen. This flexibility enables a wide range of feedstocks such as coal, waste tires, sewage sludge, biomass, municipal solid waste, plastic waste compromising moisture, volatile matter, fixed carbon, inorganic minerals/ash to be efficiently converted into value added products, including CO, H_2_, CH_4_.^2^ While coal remains the dominant global feedstock for gasification due to its cost stability and large reserves, there is increasing emphasis on utilizing renewable biomass, such as wood pellets, and nonconventional fuels like waste coal.^3^ Biomass is an essential and sustainable energy resource that can produce bio oil, syngas, and biochar as byproducts.^4^ Biomass is commonly categorized as virgin wood, energy crops, agricultural residues, food waste, and industrial waste. It is considered an ideal feedstock for gasification since it is readily available and easily transported to the desired site. Biomass typically contains lower concentrations of sulfur, nitrogen, and heavy metals than most fossil fuels, which can reduce pollutant formation during gasification.^5^ However, its application is constrained by factors such as seasonal variability, high moisture content of lignocellulosic biomass, low bulk and energy density, large storage requirement, and low quality bio oil.^6^ Also, the inconsistency biomass composition varying from one source to another is problematic.^4^ This variability underscores the need for effective feedstock characterization, particularly online characterization methods that can provide real time data on feedstock characterization, allowing operators to adjust gasifier conditions quickly to maintain optimal performance.

The cogasification of coal and biomass blends has recently attracted significant research interest due to the synergistic interactions between these two materials.^7^ ^,^ ^8^ Blending these feedstocks can reduce their overall carbon footprint and tar formation improve the H_2_/CO ratio required for liquid fuel synthesis, and leverage the inorganic matter in biomass that can catalyze coal gasification.^7^ However, the inherent heterogeneity of blended feedstocks, poses a substantial challenge to stable and reliable gasifier operation. Fluctuations in feedstock properties can trigger slagging, fouling, tar formation, sintering, and corrosion, thereby reducing overall efficiency, decreasing syngas yield, increasing maintenance requirements, and, in severe cases, leading to unplanned shutdowns or mechanical failures.^9^ In particular, variability in mineral and ash chemistry can have competing effects, some alkali and alkaline earth metals (e.g., K, Na, and Ca) may catalyze gasification reactions, whereas others (like Si and Al) may promote ash melting and cause sintering.^10^ Elevated moisture content reduces the reaction zone temperature, hindering endothermic necessary for producing H_2_ and CO reactions resulting in lower gas yield and higher tar formation.^6,11^ while increasing ash content can drastically reduce gasification efficiency and syngas production.^12^ Despite extensive research on ash related operational challenges, real time ash characterization remains a critical unmet need. Conventional post process laboratory analyses provide valuable compositional information, but the delayed feedback limits their usefulness for timely, proactive process adjustments and dynamic control.

The management of feedstock variability in gasification systems has historically been constrained by ex situ laboratory techniques, such as proximate and ultimate analysis, which typically require several days to a week for a full characterization. For example, measuring a fuel’s energy potential, commonly reported as HHV, i.e., the heat released by complete combustion (including water condensation), via adiabatic oxygen bomb calorimetry is standard. However, this method is inherently labor intensive, costly, and too time-consuming to meet the immediate data requirements of a running gasifier.^13^ As a result, these conventional approaches restrict operators to reactive feedback control, where process adjustments can only be made long after the fuel has been processed, often leading to unexpected operational failures.

To facilitate efficient feedforward control, there is an essential need for online, real time sensing that can anticipate composition changes before the material enters the reaction zone. In addition, feedforward control strategy can improve the gasifier’s efficiency and operational reliability by proactively compensating for feedstock and process disturbances. By characterizing the material in real time, controller can adjust operating conditions before deviations propagate through the reactor. This reduces fluctuations in key variables (e.g., reactor temperature profile, equivalence ratio, and steam to carbon ratio), which helps maintain stable syngas composition, consistent heating value, and high syngas yield, while also lowering the risk of instability, tar formation, and unplanned shutdowns.

LIBS has emerged as a premier analytical tool for this purpose, offering online, rapid, and multi elemental analysis with minimal sample preparation.^14^ However, the raw spectral data from LIBS is often complicated by matrix effects, saturation, spectral overlap, among other issues, which can distort the relationships between elemental concentrations and signal intensity. The integration of ML algorithms, including ANN, SVR, and RF, is critical to decode these complex “chemical fingerprints”.^15^ By utilizing ML models trained on domain based spectral features, the system can provide high accuracy predictions for parameters such as HHV, fixed carbon, and ash mineral composition instantly. This transition to a data driven, predictive framework transforms gasification from a reactive process into a resilient, automated system optimized for utilization of heterogeneous waste feedstocks.

This research presents an online and dynamic characterization approach for waste coal, wood pellets, and their blends using a combined LIBS ML framework. By processing elemental spectroscopic emissions in real time, the system provides decision quality predictions of critical gasifier feedstock parameters, including HHV, ash content, major oxides, and initial deformation temperature.

This Characterization enables proactive adjustment of operating conditions, allowing gasifier to anticipate variations in incoming feed parcels and thereby maintain a stable temperature profile, increase syngas quality, and reduce operational disturbances associated with changes in waste blend composition.

### Feedstock Characterization

Material characterization has a crucial role in evaluating the potential of and the modeling of gasification processes, since knowledge of the heating value of feedstock and its composition is essential to determine its empirical formula and stoichiometric combustion of gasification reactions. In addition, it is vital for predicting the empirical formula of volatiles to find the mass balance equation.^1^ However, these characterizations have been traditionally performed, in offline laboratories. On the contrary, real time (online) feedstock characterization would provide immediate feedback on feedstock quality, allowing for fast adjustments which can improve gasification efficiency and stability. This efficiency reflects how effectively carbon based feedstocks are converted into useful gaseous products, and it is strongly influenced by the quality and consistency of the feedstock factors that can be better managed through accurate, real time characterization.

Effective gasification performance depends on a thorough understanding of feedstock properties, as different characteristics influence syngas composition, energy yield, and process stability. Key aspects of feedstock characterization include the type of material used and its heating value, moisture content, volatile matter, fixed carbon, ash composition, mineral content, and initial deformation temperature which play an important role in determining gasification efficiency, syngas quality, and environmental impact.

Furthermore, researchers have investigated how different feedstocks, and their physicochemical properties influence gasification efficiency. For instance, Suryawanshi et al.^16^ conducted a study on five different types of feedstocks, including pine pellets, hardwood pellets, cypress mulch, pine bark chips, and corn stalk pellets to study the effect of basic physicochemical properties such as moisture content, volatile matter, ash content, higher heating value, and bulk density on a downdraft gasifier process. This research resulted in a strong correlation between feedstock composition and gasification efficiency. Specifically, materials with low moisture content, minimal ash, and high volatile matter concentrations, such as pine and hardwood pellets, demonstrated optimal performance. Conversely, feedstocks with inherently higher moisture levels, such as corn stover pellets, necessitated pre drying to mitigate efficiency losses during gasification.

Similarly, Smolinski et al.^17^ conducted studies with lignite, hard coal, and biomass at a temperature of 700 °C on steam gasification in a laboratory scale fixed bed reactor, revealing that hydrogen and carbon dioxide were the dominant components in the product gases. Lignite syngas had the highest hydrogen content, but hard coal and biomass produced comparable hydrogen concentrations. These findings highlight the role of feedstock composition in determining syngas quality and further emphasize the importance of material characterization in estimating the components of the product gases.

### Conventional Feedstock Characterization

Characterization of gasification feedstocks is a multidisciplinary task that uses chemical analyses, thermal testing, and structural studies to fully characterize a fuel. Among these analyses, proximate and ultimate analyses give the main composition, while calorific value quantifies the energy potential. Thermogravimetric analysis (TGA) provide insight into how the feedstock breaks down with heat, and mineralogical techniques like X-ray diffraction (XRD) and X-ray fluorescence (XRF) uncover the nature of inorganic matter that can dramatically affect gasifier operation.

Traditional methods for characterizing feedstocks are primarily ex situ techniques that require sample collection and transportation to a controlled laboratory environment. These approaches typically include:

Proximate Analysis: This determines the gross components of the fuel, specifically moisture content, volatile matter, fixed carbon, and ash content. Standard procedures, such as ASTM D3172 for coal, are commonly used to perform proximate analysis and ensure consistency and accuracy in the results.

Ultimate analysis determines the elemental composition of the feedstock, typically reporting the weight percentages of carbon (C), hydrogen (H), nitrogen (N), sulfur (S), and oxygen (O). Ultimate analysis methods are well established (ASTM D3176 for C, H, N, etc.) and applicable to any solid fuel.

Thermogravimetric analysis involves heating a small feedstock sample at a controlled rate (or holding it at set temperatures) in a specified atmosphere while continuously recording its weight. TGA provides a thermal decomposition profile. It can identify moisture loss, devolatilization, char oxidation, and ash yield as distinct mass loss steps. TGA is beneficial for determining kinetic parameters (activation energy, reaction order) of pyrolysis and char gasification reactions.^18^ To identify the chemical nature of volatiles responsible for TGA mass loss steps, TGA can be coupled with gas chromatography (GC) for evolved gas analysis.^19^

XRF and XRD are complementary nondestructive analytical techniques essential for characterizing the inorganic content of gasification feedstocks. XRF quantifies the elements, and XRD tells in what form they exist. XRF performs elemental analysis to determine the concentration of specific chemical elements (such as potassium (K), calcium (Ca), and silicon (Si)) by detecting characteristic X-rays emitted from excited electrons, whereas XRD identifies specific crystalline mineral phases and their structural arrangement by analyzing the interference patterns of scattered X-rays, acting as a unique fingerprint for mineral compounds. These techniques are vital for gasification because the precise composition and crystallographic form of inorganic matter (ash) dictate the feedstock’s reactivity, standard methods like ASTM D4326 are used for XRF analysis of fuel ash, where elements like potassium act as catalysts and silicon as inhibitors-and help predict operational challenges such as slagging, sintering, and agglomeration.^10^ ^,^ ^20^

While these laboratory based methods provide highly reliable data following standards, they are labor intensive and time-consuming. Full analysis can often take several days to a week, making these techniques unsuitable for the real time process optimization required in modern gasification plants. Consequently, plants often operate using feedback control, reacting to postcombustion conditions rather than proactively adjusting to feedstock variability.

### Online Feedstock Characterization

Feedstock quality variability warrants the need for online feedstock characterization and continuous measurement of key feedstock properties as the material is fed to a gasifier, allowing the gasifier to be adjusted in real time to adapt to changing feedstock conditions. Online characterization represents a fundamental transition from reactive feedback loops to proactive gasification management, enabling the implementation of feedforward control strategies.^9^

Modern systems are replacing traditional periodic laboratory analyses using spectroscopy, radiation, machine vision, and data analytics to instantly monitor feedstock quality and provides data sets continuously to plant control systems, by informing downstream processing decision, enabling automatic adjustments to keep critical parameters within target ranges.

There are important spectroscopic techniques for online feedstock analysis: Near infrared (NIR) spectroscopy with chemometric analysis is now widely used for rapid, low cost analysis, nondestructive measurements of biomass, lignocellulosic composition (cellulose, hemicellulose, and lignin) for qualitative and quantitative analysis.^21^ In addition, NIR spectroscopy is more suitable for analyzing the content of organic matter in coal, while it has a weak response to inorganic compounds, therefore, it is difficult to analyze the content of K, Ca, Na, Mg, Al, Si and other inorganic compounds in coal.^22^ Another example is using NIR spectroscopy with a partial least-squares regression model to quickly estimate key features of coal, like moisture, volatile matter, and oxygen. This model showed strong correlations between the predicted and actual values, with coefficient of determination (*R* ^2^), values of 0.97 for moisture, 0.97 for volatile matter, and 0.89 for oxygen.^23^

Alternatively, hyperspectral imaging (HSI) cameras which combine the imaging properties of a digital camera with the spectroscopic properties of a spectrometer can detect the spectral attributes of each pixel in an image and can scan a moving feed belt to map both the color/texture and spectral signature of feedstock particles. This technique enables simultaneous identification of physical chemical properties of feedstock particles.^24^ Furthermore, researchers have studied HSI integrated with principal component analysis (PCA), and ANN. While the ANN model showed good performance in predicting total phenolic compounds and chemical oxygen demand, it performed poorly in predicting total carbohydrates, cellulose, and hemicellulose^25^

Machine vision (MV) technologies have rapidly advanced as a cost-effective, scalable solution for real time biomass feedstock characterization. Using high speed digital cameras and deep learning algorithms, MV systems can continuously monitor particle characteristics such as size, shape, color, and texture. These systems detect anomalies such as particle clumping, irregular shapes, or color changes, which may indicate problems like high ash content or poor pretreatment. Research at National Renewable Energy Laboratory (NLR)^26^ has demonstrated the effectiveness of MV by training neural networks to identify normal and problematic feedstock conditions in corn stalks by correlating visual data with system performance measurements. By enabling real time, in situ monitoring feedstock variability, MV supports advanced process control strategies that reduce equipment upsets, improve feed handling reliability, and contribute to more stable and efficient biomass gasification operations.

XRF spectroscopy is a rapid and inexpensive multielement method used to determine biomass and coal characterization in seconds. It can provide detailed analysis on non destructive analysis, minimal preparation samples, simultaneous multi element quantitative and qualitative analysis. However, XRF is not suitable for atomic numbers less than 11, atomic elements like C and H which are directly related to calorific value. Therefore, combining NIR and XRF technology can provide precise analysis of coal calorific value.^22^

Prompt Gamma Activation Analysis (PGAA) is another nondestructive bulk and nuclear analytical method for determining elemental composition that detects prompt γ radiation produced by neutron capture into the atomic nucleus. In principle, every chemical element can be measured quantitatively, but with very different sensitivities. The approach is appropriate for measuring the most important components and a few trace elements (e.g., B, Cl, Co, Cd, Hg, Sm and Gd) in rocks, minerals, ceramics, glass, metals, and many inorganic materials. Especially, it has a high sensitivity for the determination of hydrogen.^27^

Raman spectroscopy is a powerful vibrational spectroscopic technique for material characterization, providing molecular and chemical information for nearly any matrix in any state of matter, in a nondestructive way, both in lab and on field conditions, by providing unique fingerprint spectra that are very useful for the identification and characterization of many compounds.^28^

Unlike traditional laboratory methods that suffer from high latency, LIBS provides rapid, in situ elemental analysis with minimal to no sample preparation, making it uniquely suited for real time monitoring of solid, liquids and gases materials.^29^ The technique relies on high energy pulsed lasers to ablate the sample surface, instantly forming a high temperature plasma that emits a “chemical fingerprint” consisting of discrete atomic and ionic emission lines.^30^ It supports both qualitative identification and quantitative measurement in a quasi-nondestructive manner.^29^ In the coal industry, LIBS is used for online quality analysis of pulverized coal on conveyor belts, determining ash content, volatile matter, and HHV within minutes.^30^ It is particularly effective for detecting light elements (e.g., Al, Li, C, H) that are difficult for other techniques like XRF to measure. When integrated with Raman using data fusion, it provides a comprehensive representation of both the elemental and molecular composition of a feedstock.^31^

Spectral analytical techniques are rapidly advancing and finding broad application for both qualitative and quantitative sample characterization. The large volumes of data produced by modern instrumentation have positioned chemometrics as a key enabling discipline, providing multivariate tools to extract the maximum chemical information from spectral data sets. In particular, LIBS, especially for direct solid analysis—has been applied across diverse matrices, including lyophilized human blood serum, plant tissues, ores, and metal alloys, as well as for the detection of contaminants in leachate and soils, compositional/deposition studies, materials analysis relevant to fusion reactor environments, coal characterization, electronic waste (e-waste) assessment, and even space exploration applications.^15^

Historically, spectroscopic techniques generally utilize chemometrics. Chemometrics uses advanced statistics and computers to design better measurement procedures and experiment conditions to extract maximum information from large raw data sets for both quantitative (regression) and qualitative (classification) analysis. Two main strategies are typically used: (1) calibration methods that build a curve between known concentrations and measured signals, and (2) calibration free methods that estimate composition without using reference materials. Within the calibration family, early work often relied on univariate regression that links a single emission line to concentration. This approach breaks down for complex matrices where lines overlap, and matrix effects distort intensities. Consequently, multivariate calibration that leverages the full spectrum is preferred. Linear methods such as partial least-squares (PLS) and least-squares (LS) regression address many cases, but for nonlinear or noisy data sets, machine learning models tend to capture structure better and deliver more stable predictions.^32^

Machine learning is gaining importance as its differences from traditional chemometric methods become more apparent. Unlike simple linear models, ML methods can model complex, nonlinear relationships and handle large, high dimensional data sets without restricting the analysis to specific functional groups or requiring extensive prior knowledge. Moreover, ML is learning from data while chemometric methods mainly based on mathematical and statistical models. Finally, ML algorithms utilize traditional chemometrics and statistical methods as a useful tool.^33^

By continuously and in situ monitoring and adjusting for feedstock variability, facilities can enhance gasifier performance, efficiency, and syngas production. The combination of spectroscopy and artificial intelligence with real time analytics enables a shift from static operation to a smart, adaptive process. Thus, operators can run gasifiers more efficiently and safely, knowing that any deviation in feed quality will be immediately detected and compensated for.

## Methods

This Section outlines the experimental methodology used to generate a traceable data set which links LIBS measurements with standardized laboratory reference characterization for heterogeneous gasifier feedstocks, with an emphasis on controlled handling and repeatable measurement. The section first summarizes the sample preparation strategy and the corresponding laboratory analyses used to establish the ground truth target properties. It then describes the conveyor based LIBS instrumentation and operating conditions, followed by the data acquisition and quality control steps implemented to ensure stable and comparable spectra across the sample set.

### Sample Matrix and Preparation

To train machine learning regression models for online LIBS based prediction of gasifier relevant feedstock properties, an experimental matrix was designed to impose control, interpretable variability in both composition and particle size. The matrix comprised four different feedstocks (two waste coals and two biomass types) as shown in Figure 1 and four different blended formulations. Each blend was prepared at fixed mass ratios Table 1 and evaluated at two particle size conditions (1–3 mm and under 1 mm), yielding 12 groups.

**Figure 1:**
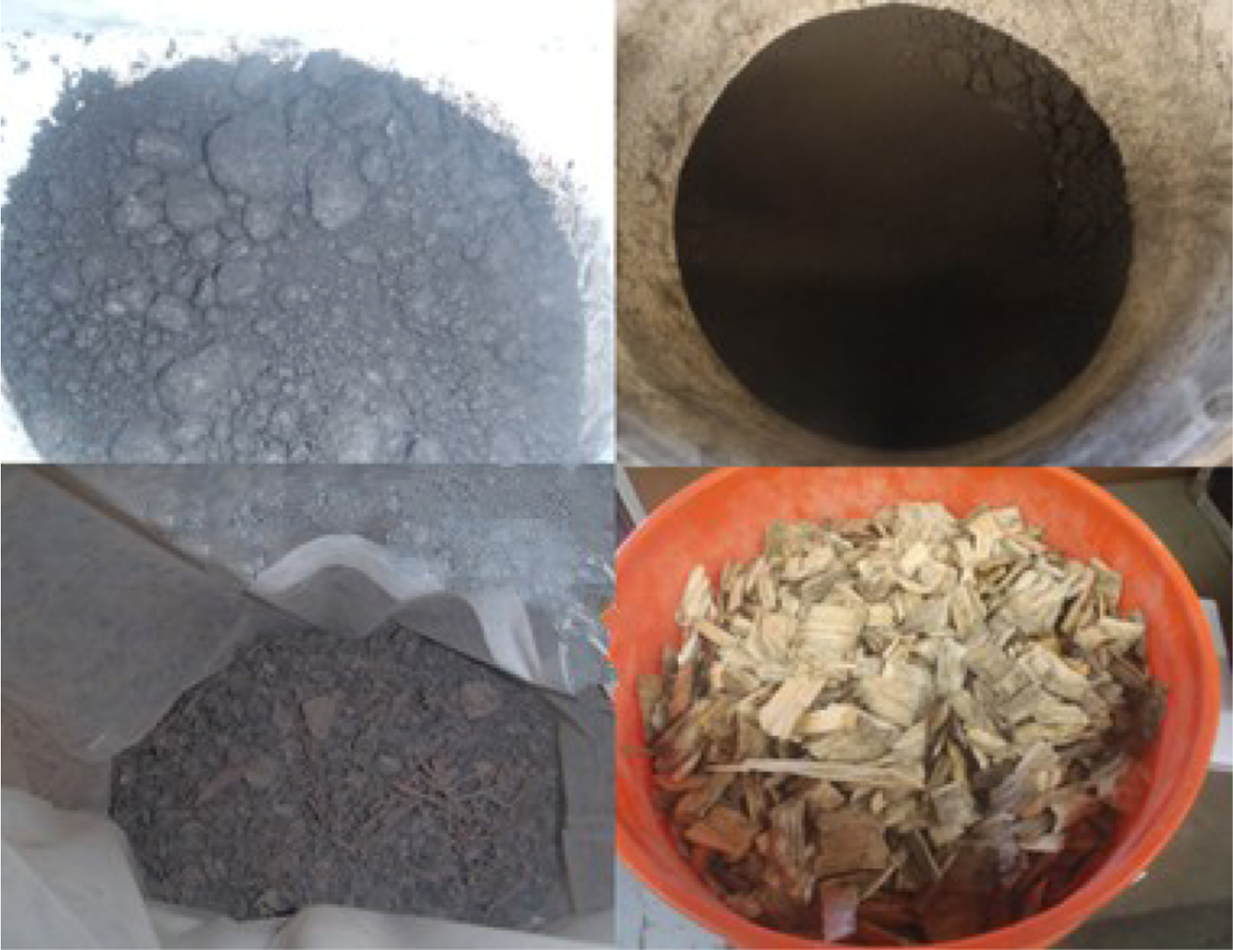
Feedstocks (waste coal 1 (top left), waste coal 2 (top right), biomass II (vineyard–bottom left), biomass I (bottom right)).

**Table 1:** Sample Matrix Blending Information

sample name	substance 1	ratio (%)	substance 2	ratio (%)	blend size
Feedstock-1	biomass-I	100.00	–	0.00	–
Feedstock-2	biomass-II	100.00	–	0.00	–
Feedstock-3	waste coal-I	100.00	–	0.00	–
Feedstock-4	waste coal-II	100.00	–	0.00	–
Blend-1.1	biomass-I	75.00	waste Coal II	25.00	2 mm
Blend-1.2	biomass-I	75.00	waste Coal II	25.00	500 μm
Blend-2.1	biomass-I	95.00	waste Coal I	5.00	2 mm
Blend-2.2	biomass-I	95.00	waste Coal I	5.00	500 μm
Blend-3.1	biomass-I	99.00	waste Coal I	1.00	2 mm
Blend-3.2	biomass-I	99.00	waste Coal I	1.00	500 μm
Blend-4.1	blend-1	93.50	limestone	6.50	2 mm
Blend-4.2	blend-1	93.50	limestone	6.50	500 μm

Mechanical processing of the feedstocks involved a multi stage reduction sequence to ensure uniformity and surface consistency. Initial size reduction was achieved using an industrial shredder and a specialized crushing machine, followed by further refinement via a manual mortar and pestle. To categorize the materials into the required size groups (e.g., 1–3 mm and below 1 mm), the ground samples were processed through a mechanical shaker for precise sieving. For the composite groups as shown in Figure 2, the constituents were integrated according to specific mass ratios (as detailed in Table 1) and homogenized using a concrete mixer to ensure a uniform distribution of coal and biomass particles throughout the volume.

**Figure 2:**
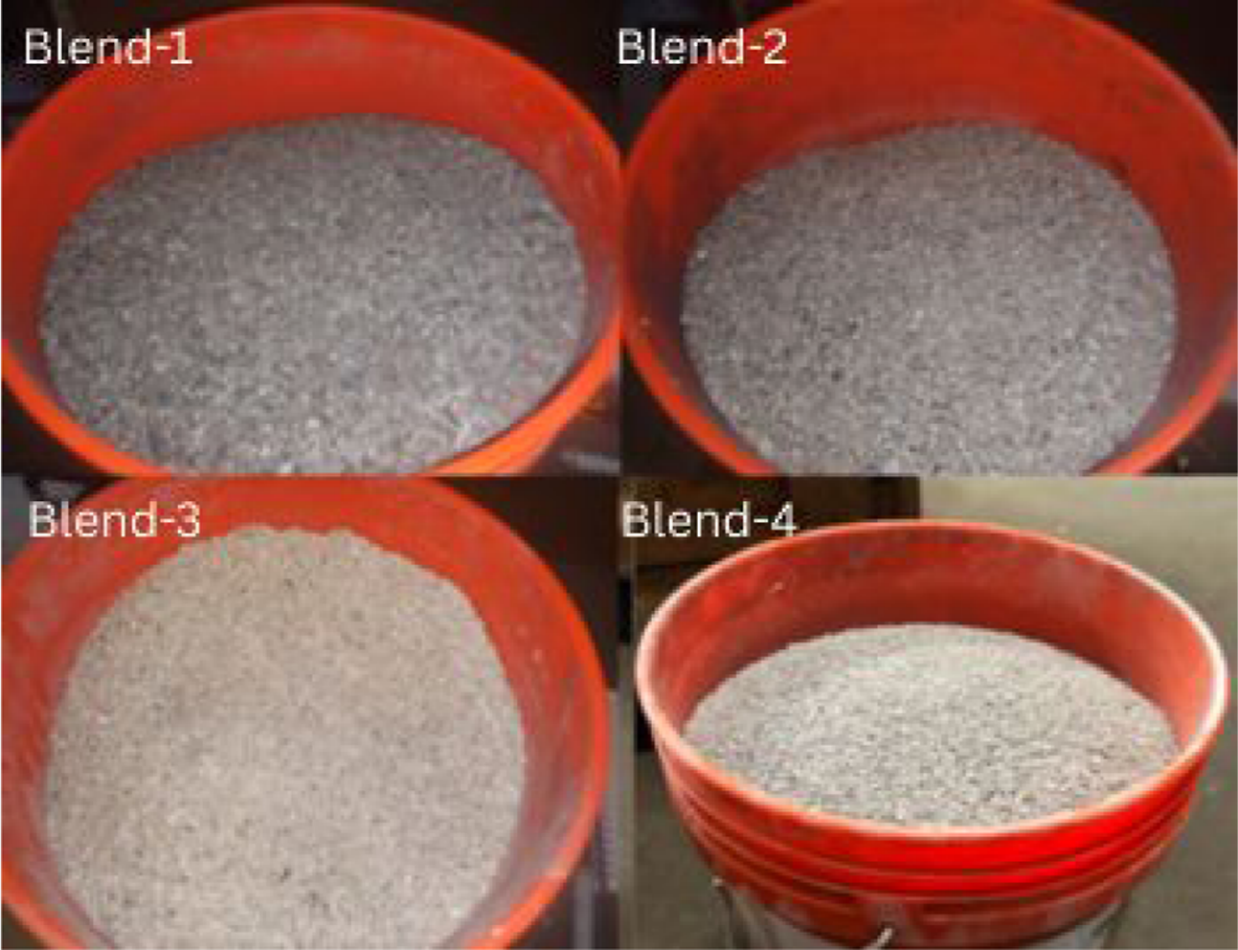
Blended materials (blend 1-blend 2-blend 3-blend 4).

Following preparation, representative samples of each group were submitted to an accredited laboratory to determine the fundamental chemical and thermal properties required for training the ML models. These target variables provided the ground-truth data necessary for quantitative measurement and included:

- Proximate and Ultimate Analysis: Including total ash content (%) (ASTM D3174-12), fixed carbon (%) (ASTM D3172-13), gross heating value (moisture and ash free basis-MAF) (ASTM D5865), and total sulfur (%) (ASTM D4239).
- Inorganic and Alkali Composition: Detailed quantification of oxide concentrations, including SiO_2_, Al_2_O_3_, TiO_2_, Fe_2_O_3_, CaO, MgO, K_2_O, Na_2_O, as well as the initial deformation (softening) temperature (ASTM D1857).

This structured approach ensured that the resulting data set encompassed a wide spectrum of physicochemical variabilities, providing a robust foundation for the subsequent development of adaptive, high-efficiency predictive models for gasifier control.

Table 2 compiles a statistical summary of the laboratory “ground truth” measurements used to train and validate the LIBS machine learning models. It reports, for each feedstock category and engineered blend in the sample matrix, both the central tendency and the within sample variability (mean and standard deviation) of key gasifier relevant properties. These targets include proximate/ultimate type metrics (ash, moisture and ash free heating value, total sulfur, and fixed carbon), an overall alkali content indicator, and the initial deformation temperature, all determined by standardized laboratory methods.

**Table 2:** Statistical Summary of Laboratory Results of the Sample Matrix (Proximate Analysis and Other Parameters)

						alkali contents **(%)** **(ASTM D3174–12)**								
**unique sample name**	**Stat**	total Ash**(%)** **(ASTM D3174–12)**	heating value MAF **(BTU/lbm) (ASTM D5865)**	total sulfur **(%)** **(ASTM D4239)**	fixed carbon **(%)** **(ASTM D3172–13)**	**SiO**_**2**_	**Al**_**2**_ **O**_**3**_	**TiO**_**2**_	**Fe**_**2**_ **O**_**3**_	**CaO**	**MgO**	**K**_**2**_ **O**	**Na**_**2**_ **O**	initial deformation temperature **(°C)** **(ASTM D1857)**
Biomass-I	std	0.05	21.80	0.00	1.45	0.14	0.06	0.00	0.00	0.21	0.28	0.30	0.06	10.61
	mean	0.66	9098	0.06	14.43	15.50	5.99	0.28	4.02	28.55	12.20	8.71	1.65	1333.61
Waste Coal-I	std	0.19	9.79	0.01	0.14	1.27	0.21	0.00	0.11	0.04	0.01	0.03	0.02	0.79
	mean	51.02	13993	0.44	30.49	60.10	22.05	1.16	5.95	4.61	1.76	4.07	0.44	1276.67
Waste Coal-II	std	0.30	8.03	0.00	0.39	0.69	0.07	0.04	0.08	0.17	0.02	0.10	0.05	4.32
	mean	27.24	12794	0.88	48.47	51.38	25.80	1.85	4.04	9.62	1.91	0.90	0.69	1314.17
Biomass-II	std	1.25	15.87	0.04	0.89	1.23	0.49	0.02	0.13	0.24	0.11	0.18	0.01	16.50
	mean	19.38	9995	0.07	23.33	53.80	11.62	0.61	4.32	5.31	1.73	17.29	0.86	1312.78
Blend 1.1	std	0.07	121.70	0.01	0.37	0.66	0.20	0.00	0.03	0.04	0.04	0.11	0.02	23.96
	mean	32.96	11380	0.25	26.49	54.60	21.40	1.26	6.37	5.68	2.07	4.03	0.60	1243.61
Blend 1.2	std	0.15	67.52	0.01	0.16	0.61	0.21	0.00	0.03	0.06	0.04	0.11	0.02	9.82
	mean	28.82	11891	0.20	30.43	50.60	21.90	1.29	7.78	7.60	2.29	3.90	0.56	1243.61
Blend 2.1	std	0.39	69.78	0.01	0.04	0.43	0.23	0.00	0.00	0.08	0.06	0.07	0.06	14.93
	mean	8.99	9966	0.24	26.97	46.50	24.80	1.71	1.84	10.50	2.84	1.72	1.61	1326.67
Blend 2.2	std	0.13	57.69	0.01	0.25	0.45	0.20	0.00	0.00	0.09	0.06	0.08	0.04	42.43
	mean	7.36	9693	0.21	24.72	47.80	21.60	2.02	4.83	11.20	2.51	2.07	0.89	1256.67
Blend 3.1	std	0.20	118.36	0.01	0.58	0.61	0.07	0.04	0.03	0.16	0.04	0.32	0.08	6.68
	mean	2.79	9184	0.07	18.87	45.70	23.30	1.79	1.79	9.39	3.61	2.96	1.16	1268.06
Blend 3.2	std	0.11	61.67	0.00	0.17	0.59	0.06	0.04	0.07	0.22	0.04	0.48	0.08	34.96
	mean	2.11	9138	0.08	18.80	44.60	20.00	1.72	3.80	12.70	3.45	4.38	1.12	1205.28

### LIBS Experiment Setup

LIBS focuses a high energy laser pulse on a sample, ablating a microscopic layer of sampling material and creating a short live plasma. As the plasma cools, excited atoms and ions emit characteristic light that reveals the sample’s elemental composition. This emission is collected via a fiber optic cable and dispersed by a spectrometer to produce an intensity versus wavelength spectrum.

The experimental architecture for this study utilized a high precision LIBS system configured for the analysis of heterogeneous waste streams. The setup, illustrated in Figure 3, integrated advanced laser optics with a high resolution detection assembly and a dynamic transport system.

**Figure 3:**
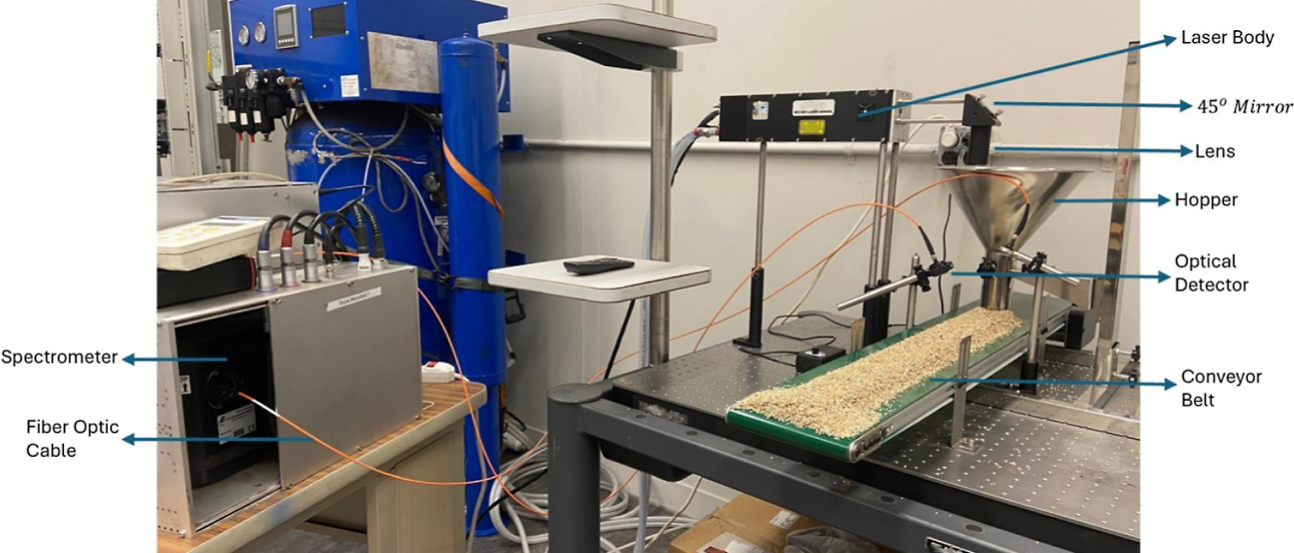
Lehigh University—LIBS experiment setup.

The primary irradiation source consisted of a pulsed Class IV, lamp pumped solid state Nd/YAG laser (Lumibird) operating at wavelength of 1064 nm. This Q-switched system was configured to deliver laser pulses with an energy of approximately 300 mJ and a characteristic 6 ns pulse duration. The laser beam was directed through a 45 deg mirror and focused vertically onto the feedstock surface using a lens with a 100 mm focal length. This configuration produced a laser footprint on the sample surface of approximately 300 μm in diameter. To ensure maximum spectral reliability and reduce ambient interferences common in waste material analysis, the interaction region was maintained under an argon atmosphere.^34^

Optical emission from the resulting transient micro plasma was captured by a lens system and coupled into a fiber optic cable for transmission to the spectrometer. The system utilized an Echelle spectra analyzer (ESA) (LLA Instruments GmbH) capable of providing high spectral resolution between 0.02 and 0.06 nm. Due to the inherent limitations of the primary Echelle system in capturing wavelengths above 660 nm, an auxiliary spectrometer was integrated into the assembly specifically to enable the detection of potassium (K) emission lines. The signals were recorded by an intensified charge coupled device (ICCD) detector. To optimize the signal-to-noise ratio (SNR), the ICCD was operated with a 1 μs gate delay and a 5 μs integration (gate) time.

### Data Acquisition and Quality Control

To enhance the reliability, each representative spectrum was formed by averaging 800 consecutive laser shots, which increases the SNR, defined as the magnitude of the analytical signal relative to the baseline noise level, calculated with peak signal level is divided by standard deviation of the noise and reduces the RSD (relative standard deviation), defined as the standard deviation of repeated measurements normalized by their mean, typically reported as percentage. This acquisition was performed dynamically as the feedstock materials were flown across a conveyor belt at a constant speed. Operating at a repetition frequency of 2 Hz, the system required 400 s to acquire the raw spectrum and extract a single spectral feature vector. While this duration ensures a stable spectral signature for heterogeneous specimens, it remains significantly more time efficient than conventional offline laboratory characterization technique and providing a robust data set for subsequent machine learning regression. Each group was characterized by 20 independent LIBS observations. This structured acquisition protocol resulted in a library of 240 spectral feature vectors which primarily consists of 9 prominent wavelengths corresponding to C, Fe, Mg, Si, Al, Ti, Ca, Na, and K, providing high quality foundation for subsequent machine learning regression and feedstock characterization.

Following data collection and initial preprocessing, the quality of the LIBS wavelength intensity data was assessed via RSD in Table 3, computed as the ratio of the standard deviation to the mean across replicate spectra for each group. For each spectral line or designated feature set, an RSD threshold of 15% was utilized to confirm acceptable repeatability. Any values exceeding this 15% limit were flagged to trigger a rigorous review of the acquisition and preprocessing parameters. Overall, the data set exhibited a good short-term stability: 96 of 108 RSD values (88.9%) are ≤15%, indicating acceptable repeatability for most species and samples as seen in Table 3.

**Table 3:** RSD of Selected Spectral Lines

RSD	C %	Fe %	Mg %	Si %	Al %	Ti %	Ca %	Na %	K %
blend 01 2 mm	16.54	7.72	9.18	5.24	3.96	7.07	7.10	10.03	25.61
blend 01 500 μm	7.63	4.50	3.45	2.22	2.65	4.97	3.75	5.01	3.34
blend 02 2 mm	6.25	6.73	2.20	2.60	4.25	6.42	5.43	8.77	2.24
blend 02 500 μm	16.98	12.00	3.11	3.52	4.28	3.89	5.39	8.30	9.84
blend 03 2 mm	4.74	12.10	10.44	13.26	13.60	14.34	14.29	16.05	3.47
blend 03 500 μm	10.46	8.50	3.19	10.54	14.71	13.55	7.39	27.68	9.04
blend 04 2 mm	14.82	13.00	4.26	12.38	12.59	13.55	2.53	11.80	3.60
blend 04 500 μm	23.10	23.80	17.79	14.64	10.82	14.48	4.03	18.71	9.81
waste Coal–II	9.95	5.13	3.52	2.40	2.44	4.18	4.24	3.79	8.23
biomass-I	5.29	11.41	3.13	12.33	11.34	20.09	5.55	21.06	19.00
waste Coal–II	10.15	7.91	4.58	3.87	3.95	5.86	1.73	3.51	2.57
biomass-II	6.95	5.40	1.78	2.19	3.43	4.56	2.57	12.14	1.66

### Machine Learning Methods

Despite its status as a versatile tool for real time, in situ elemental analysis, LIBS is hindered by several significant analytical challenges, notably matrix effects, self-absorption, and spectral interference.^15^ Matrix effects occur when the physical properties and chemical composition of a sample uniquely influence the laser matter interaction, causing signal variations even at constant analyte concentrations. Self-absorption further complicates quantitative accuracy by lowering the maximum intensity of spectral lines, which results in nonlinear intensity concentration relationships. Furthermore, poor shot to shot reproducibility frequently arises from the transient and inhomogeneous nature of plasma evolution and fluctuations in laser energy.

To mitigate these disadvantages, modern research increasingly integrates ML and deep learning (DL) algorithms to extract meaningful patterns from complex, high dimensional spectral data. Supervised models such as Support Vector Machines (SVM), RF, and ANN regression are employed to enhance predictive accuracy and robustness against matrix interferences by learning hidden underlying relationships.

In previous studies on the quantitative characterization of complex heterogeneous feedstocks via LIBS, various ML regression algorithms have been exploited to overcome severe matrix effects and improve prediction accuracy for key process parameters. For instance, regarding coal analysis, researchers^14^ demonstrated that advanced regression techniques, such as partial least squares (PLS) combined with variable selection, could significantly reduce root mean square error of prediction (RMSEP) for fixed carbon and ash content. Furthermore, the potential of deep learning for quantitative tasks in metallic recycling was highlighted.^15^ In that study, convolutional neural networks (CNNs) were used to quantify alloying elements in aluminum scrap, achieving lower prediction errors than traditional random forests and logistic regression models.

Building on this motivation, the present work developed supervised ML regression models to estimate key feedstock properties directly from LIBS measurements for rapid, online material characterization. Model development and evaluation were implemented in Python using established libraries, like scikit learn.

### Data Normalization and Validation

All input features were normalized prior to model fitting, because LIBS predictors (e.g., emission line intensities and derived indices) span different numerical scales and can exhibit large dynamic ranges. Specifically, predictors were standardized using z-score scaling (zero mean and unit variance), with scaling parameters estimated only from the training data within each split and then applied to the corresponding validation/test data. This fold wise normalization improves numerical conditioning, prevents high magnitude variables from dominating the learning process, and maintains strict separation between training and evaluation data (i.e., no information leakage). When required for targets with broad ranges, an analogous training fold transformation was applied to the response variable and subsequently inverted so that all reported results remain in physically interpretable units.

### Feature Extraction

Predictor variables were extracted from the LIBS spectra prominent wavelength as tabulated numerical features. In this study, each sample was represented by a fixed length feature vector composed of preselected LIBS emission line intensities (i.e., intensity values at specific wavelengths corresponding to relevant atomic/ionic transitions). These line intensity features were augmented with engineered spectral descriptors (e.g., ratios or combined indices derived from selected lines) to capture compositional relationships that are not fully represented by individual intensities. The formulation used six derived engineering spectral parameters: silica value, base, acid, R250, dolomite ratio, and base-to-acid ratio. R250 is related to slagging tendency through deposit viscosity. These parameters were calculated as follows: silica value = Si/(Si + Fe + Ca + Mg), base = Fe + Ca + Mg + K + Na, acid = Si + Al + Ti, R250 = (Si + Al)/(Si + Al + Fe + Ca), dolomite ratio = (Ca + Mg)/(Fe + Ca + Mg + K + Na), and base/acid ratio = (Fe + Ca + Mg + K + Na)/(Si + Al + Ti).^35^

Different feature sets were defined as a priori as explicit column lists for each target property. For each target, an identical modeling and validation pipeline was applied across all candidate predictor variable sets (i.e., each alternative LIBS feature set) under the same training and evaluation conditions. This controlled framework enables a direct assessment of which LIBS-derived representations provide the most informative predictors for material characterization.

### Machine Learning Models and Hyperparameter Optimization

To capture potentially nonlinear relationships between LIBS derived features and material properties, three complementary regression families were evaluated: multilayer perceptron regression (MLP), random forest regression, and support vector regression.

The SVM constructs a hyperplane or a set of hyperplanes in a high dimensional feature space to separate data classes. The optimal hyperplane is defined as the linear decision function that maximizes the margin between the vectors of two different classes.^36^ SVR adapts the margin maximization principle of SVM classification to regression problems. Unlike classification, which seeks to separate classes with the widest possible margin, SVR aims to fit as many instances as possible on the margin while limiting margin violations. The width of this margin is controlled by the hyperparameter ϵ, making the model ϵ insensitive: adding training instances within the margin does not affect the model’s predictions.

The training objective is to minimize the model’s complexity (represented by the weight vector, **w**) and the prediction errors (represented by slack variables, ξ). This is formulated as the primal problem

1 $$\min\left(\frac{1}{2}w^{T}w+C\sum_{i=1}^{m}\left(\xi_{i}+\xi_{i}^{*}\right)\right)$$

Subject to the constraints

2 $$y_{i}-\left(w^{T}\Phi \left(x_{i}\right)+b\right)\leq \epsilon +\xi_{i}$$

3 $$\left(w^{T}\Phi \left(x_{i}\right)+b\right)-y_{i}\leq \epsilon +\xi_{i}^{*}$$

4 $$\xi_{i},\xi_{i}^{*}\geq 0$$

Where:

-
$\frac{1}{2}w^{T}w$ penalizes model complexity to ensure flatness.
- *C* is the regularization hyperparameter balancing the trade-off between model complexity and tolerance for margin violations.
- ξ_*i*_ and ξ_i_^*^ are slack variables representing deviations exceeding the margin ϵ.

To handle nonlinear regression tasks, SVR utilizes a mathematical technique known as the “kernel trick.” This allows the algorithm to implicitly map inputs to high-dimensional spaces without explicitly computing the transformation Φ­(*x*). The algorithm solves the dual problem rather than the primal problem, which enables the solution to be expressed as a linear combination of a subset of training points called support vectors.^37^

The prediction for a new instance is computed using the kernel function, which evaluates the dot product between the input vector and the support vectors in the high-dimensional feature space

5 $$f\left(x\right)=\sum_{i=1}^{m}\left(\alpha_{i}-\alpha_{i}^{*}\right)K\left(x_{i},x\right)+b$$

Where:

- *K*(*x*_*i*_, *x*) is the kernel function (e.g., Gaussian RBF).
- α_*i*_ and α_i_^*^ are Lagrange multipliers found by maximizing the dual objective function.
- *x*_*i*_ represents the support vectors (a subset of training data).
- *b* is the bias term.

ANN consists of layers of interconnected processing units (neurons), organized into input, hidden, and output layers. For a given neuron *j* in layer *l*, the input *z*_*j*_^(*l*)^ is the weighted sum of the activation from the previous layer (*l* −1) plus a bias term *b*

6 $$z_{j}^{\left(l\right)}=\sum_{k}w_{jk}^{\left(l\right)}a_{k}^{\left(l-1\right)}+b_{j}^{\left(l\right)}$$

The output activation *a*_*j*_^(*l*)^ is then computed by applying a non linear activation function σ (such as hyperbolic tangent (Tan h) and the rectified linear unit (ReLU)) to this sum

7 $$a_{j}^{\left(l\right)}=\sigma \left(z_{j}^{\left(l\right)}\right)$$

Where *w*_*j*k_^(*l*)^ is the weight connecting neuron *k* in the previous layer to neuron *j* in the current layer, *a*_*k*_^(*l*–1)^ is the activation output from the previous layer, σ is the activation function.^31^ The network parameters (weights and biases) are optimized by minimizing a cost function *E*(**w**), which quantifies the discrepancy between the model’s predictions and the ground truth, such as the mean squared error (MSE) for regression. The MSE cost function is expressed as

8 $$E\left(w\right)=\frac{1}{N}\sum_{j=1}^{N}{\left(a_{j}^{L}-y_{j}\right)}^{2}$$

Where *a*_*j*_^*L*^ represents (where *L* is the final layer) prediction value, *y*_*j*_ is ground truth, and *N* represents the number of samples (or batch size). Model parameters (weights **w** and biases *b*) are optimized using backpropagation. This algorithm computes the partial derivative of the cost function with respect to each parameter using the chain rule, allowing for iterative updates via gradient descent.^31^

9 $$\frac{\partial E}{\partial w_{jk}^{\left(l\right)}}=\frac{\partial E}{\partial z_{j}^{\left(l\right)}}\times \frac{\partial z_{j}^{\left(l\right)}}{\partial w_{jk}^{\left(l\right)}}=\delta_{j}^{\left(l\right)}a_{k}^{\left(l-1\right)}$$

δ_*j*_^(*l*)^ represents the local error term (or local gradient) associated with neuron *j* in the current layer *l*. Mathematically, it is the partial derivative of the cost function *E* with respect to the weighted input *z*_*j*_^(*l*)^ of that neuron.

10 $$\delta_{j}^{\left(l\right)}=\frac{\partial E}{\partial z_{j}^{\left(l\right)}}$$

This term quantifies how much a change in the total input to neuron *j* affects the total error of the network. *a*_*k*_^(*l*–1)^ represents the output activation of neuron *k* in the previous layer (*l* −1). This value serves as the input to the weight *w*_*jk*_ connecting to neuron *j*. The product of these two terms represents the gradient of the cost function with respect to the specific weight *w*_*jk*_. This relationship is derived using the chain rule of calculus.

Random Forest Regression is an ensemble method that constructs a collection of regression trees. Unlike classification, which uses voting, the forest predictor for regression is formed by taking the average of the individual tree predictors.^38^

The algorithm uses the classification and regression tree (CART) approach, which splits the training set to minimize the mean squared error (MSE) rather than impurity measures like Gini or entropy which are standard in classification. The objective is to find the pair of features *k* and threshold *t*_*k*_ that minimizes the following cost function^37^

11 $$J\left(k,t_{k}\right)=\frac{m_{left}}{m}{MSE}_{left}+\frac{m_{right}}{m}{MSE}_{right}$$

Where:

- *J*(*k*, *t*_k_) is the cost function for splitting.
- *m*_left/right_ is the number of instances in the left/right subset created by the split.
- *m* is the total number of instances in the node.
- MSE_node_ is the mean squared error within a specific node, defined as^37^

12 $${MSE}_{node}=\frac{1}{m_{node}}\sum {\left(y_{node}-y^{\left(i\right)}\right)}^{2}$$

where:

- *y* ^(*i*)^ is the actual target value for the *i*-th instance in the node.
- *y*_node_ is the predicted value for the node, typically the average of the target values of the training instances in that node.

As the number of trees approaches infinity, the mean squared generalization error converges to a limit, ensuring the model does not overfit. The generalization error depends on the correlation between the residuals of the trees and the mean squared error of the individual trees; lower correlation and lower individual tree error lead to better forest performance.

Weighted ensemble methods combine predictions from multiple learners to create a “strong” learner. In such algorithms, predictors are trained sequentially. Each predictor is assigned a weight based on its accuracy; predictors that perform well are given more influence in the final decision. The weights are updated iteratively to minimize the overall error rate.

Model complexity was selected using a nested cross validation (nested CV) strategy to ensure unbiased performance estimation. In this framework, an outer CV loop was used to estimate generalization performance, while an inner CV loop was used exclusively for hyperparameter tuning and model selection. Hyperparameters were optimized via Bayesian optimization within the inner loop, and the selected configuration was then refit on the corresponding outer training subset and evaluated on the outer test fold.

For the multilayer perceptron (MLP), the search space included the number of hidden layers (1–5), the number of neurons per hidden layer (10–200), and the activation function (logistic, tanh, or ReLU). For the random forest (RF), the tuned parameters comprised the number of trees (50–300), maximum tree depth (2–15), the feature-subsampling rule (square-root or log2), minimum samples required to split a node (2–10), and minimum samples per leaf (2–5). For the support vector regressor (SVR), the search space included the kernel family (linear, polynomial, radial-basis function, or sigmoid), the regularization parameter (0.1–100), the polynomial degree (1–5, when applicable), and the insensitive-loss width (0.001–1.0).

In addition to tuning the base learners, the ensemble combination was treated explicitly by optimizing non-negative weights assigned to each base model over the range 0–1 and then normalizing the weights to sum to one, yielding a convex weighted ensemble. The nonnegative ensemble weights were determined by Bayesian optimization inside the inner repeated cross-validation loop. After the MLP, RF, and SVR base models were tuned, the code optimized three ensemble-weight variables against the inner-CV RMSE objective, then used the best weight triplet to build the Voting Regressor.

To support reproducibility, the remaining model settings were held constant and are reported explicitly. The MLP was trained with a fixed convergence tolerance and maximum iteration cap, with early stopping disabled to ensure deterministic training behavior under the stated iteration limit. The RF was trained with a fixed random seed and parallel tree construction, while the SVR used the tuned kernel and regularization parameters with other settings fixed at their standard values. All models were embedded in a leakage-controlled preprocessing framework in which feature scaling and (when used) target scaling were learned only from the corresponding training partitions within cross-validation and then applied to the held-out data, preventing information leakage. Robustness is supported by two design elements: (i) the heterogeneous ensemble combines complementary inductive biases (nonlinear neural network, bagged decision trees, and margin-based regression), reducing sensitivity to any single model’s failure mode; and (ii) the model selection process relies on nested cross-validation with an independent external test set, which reduces tuning optimizm and provides stable performance estimates across multiple train/test partitions.

### Machine Learning Evaluation and Validation Strategy

Model performance was assessed using leakage controlled design that combined nested cross validation (CV) with an independent external test set. The data set was first partitioned into (i) a training subset used for model development and (ii) an external hold out subset comprising 20% of the samples, which was reserved for final testing. Within the training subset, model development followed a nested 5-fold CV strategy with explicit repetition in the inner loop, the outer loop used a single 5-fold CV partition to estimate generalization performance, while the inner loop used 5-fold CV repeated twice, and was used exclusively for hyperparameter tuning (and ensemble weight optimization when applicable). For each outer split, the optimized model was refit on the corresponding outer training fold and evaluated on the held out outer test fold, providing unbiased out of fold predictions. After completing nested CV, the selected model configuration was refit on the full training subset and evaluated once on the external hold out set to independent estimate of generalization performance.

Prediction accuracy was quantified using the root-mean-square error (RMSE) and the coefficient of determination (*R* ^2^). For comparison across targets with different magnitudes, errors were additionally expressed in relative terms (percent based RRMSE).

1 **Coefficient of determination (** *R* ^**2**^ **):** *R* ^2^ reflects how well the model explains the variability in the observed data; values closer to 1 indicate a stronger fit. However, *R* ^2^ by itself does not always capture predictive accuracy.^39^

13 $$R^{2}=1-\frac{\sum_{i=1}^{n}{\left(y_{i}-{ŷ}_{i}\right)}^{2}}{\sum_{i=1}^{n}{\left(y_{i}-\mathrm{y̅}\right)}^{2}}$$

2 **Root mean square error (RMSE):** RMSE summarizes the typical magnitude of prediction errors and is widely used in regression, particularly when errors are assumed to be approximately normally distributed^39^

14 $$RMSE=\sqrt{\frac{1}{n}\sum_{i=1}^{n}{\left(y_{i}-{ŷ}_{i}\right)}^{2}}$$

3 **Relative root-mean-square error (RRMSE):** RRMSE normalizes RMSE by the mean of the observed values, producing a dimensionless error measure that enables comparisons across models or data sets with different scales. Model performance can be interpreted as excellent when RRMSE <10%, good for 10–20%, fair for 20–30%, and poor above 30%.^40^

15 $$RRMSE=\frac{\sqrt{\frac{1}{n}\sum_{i=1}^{n}{\left(y_{i}-{ŷ}_{i}\right)}^{2}}}{\mathrm{y̅}}\times 100$$

This evaluation strategy provides an unbiased estimate of predictive performance and supports reliable comparison among LIBS feature sets and ML model families for material characterization.

To visually evaluate agreement between measurements and predictions, parity plots (predicted vs measured) were generated using predictions on the independent external test set. Parity plots included the 1:1 reference line to represent ideal agreement and ±10% tolerance bands (0.9× Measured and 1.1× Measured) to provide an interpretable acceptance window for practical LIBS based material characterization. These plots were used to evaluate bias (systematic deviation from the 1:1 line), dispersion across the prediction range, and the presence of outliers, enabling direct comparison of individual models and the weighted ensemble under the same validation protocol.

## Machine Learning Results and Discussion

### Machine Learning Model Performance Comparison

Data preprocessing and model development was performed using the scikit-learn machine learning library in the Python programming environment. Four models were evaluated for their predictive performance with respect to relevant material composition and gasification parameters from the proximate and ultimate analyses, including the fixed carbon, total ash, and HHV, initial deformation temperature, and major ash forming oxides (SiO_2_, Al_2_O_3_, Fe_2_O_3_, CaO, MgO, K_2_O, Na_2_O), which can have direct implications for process optimization and slag management of gasification. The performance of the trained models has been evaluated using the *R* ^2^, RMSE and RRMSE of external test set and inner RMSECV (root-mean-square error of cross validation. Apart from the *R* ^2^ metric, lower values of these metrics indicate better model performance. For clarity, we note that the *R* ^2^ is a unitless metric, RMSE RMSECV are expressed in the same units as the target variable, and RRMSE is presented as a percentage (%), allowing for scale independent comparisons.

Table 4 summarizes the predictive performance of the three base regressors (MLP/ANN, RF, SVR) and a weighted ensemble for proximate/ultimate properties (HHV, ash, fixed carbon, sulfur) and major ash forming oxides, together with the initial deformation temperature.

**Table 4:** Cross-Validation Performance Metrics for the Predicted Process Parameters of Interest

					alkali contents							
**model/parameter**	**HHV MAF** **(BTU)**	**ash, DB** **(%)**	**fixed carbon, DB** **(%)**	**sulfur, DB**	**SiO_2_** **(%)**	**Al**_**2**_ **O**_**3**_ **(%)**	**Fe**_**2**_ **O**_**3**_ **(%)**	**CaO** **(%)**	**MgO** **(%)**	**K**_**2**_ **O** **(%)**	**Na**_**2**_ **O** **(%)**	**fluid T.** **(%)**
***R*** ^**2**^												
MLP	0.98	0.99	0.98	0.97	0.98	0.99	0.99	1.00	0.99	0.99	0.94	0.79
RF	0.95	0.98	0.96	0.96	0.92	0.93	0.98	0.94	0.96	0.96	0.95	0.76
SVR	0.98	0.99	0.97	0.97	0.97	0.96	0.96	0.99	0.96	0.97	0.93	0.71
ensemble	0.98	0.99	0.98	0.97	0.98	0.98	0.99	1.00	0.99	0.99	0.94	0.79
**RMSE**												
MLP	209.99	1.58	1.15	0.04	1.65	0.59	0.17	0.41	0.25	0.47	0.09	22.90
RF	354.97	2.03	1.70	0.05	3.07	1.45	0.25	1.45	0.54	0.86	0.09	24.70
SVR	227.36	1.51	1.51	0.04	1.98	1.03	0.34	0.68	0.58	0.77	0.10	26.05
ensemble	205.74	1.25	1.17	0.04	1.66	0.59	0.17	0.41	0.25	0.47	0.09	23.12
**RRMSE (%)**												
MLP	1.92	7.45	4.25	15.52	3.41	2.99	3.41	4.26	7.71	9.26	10.58	1.64
RF	3.24	9.57	6.27	19.09	6.33	7.28	5.03	15.06	17.11	16.87	10.25	1.77
SVR	2.07	7.14	5.55	15.77	4.08	5.18	6.83	7.05	18.39	15.01	11.87	1.94
ensemble	1.88	5.90	4.31	15.22	3.42	2.99	3.40	4.26	7.71	9.28	10.38	1.66
**inner RMSECV**												
MLP	214.49	1.76	1.58	0.03	2.03	0.74	0.26	0.89	0.37	0.61	0.13	21.36
RF	320.43	2.79	2.14	0.04	3.88	2.00	0.41	2.06	0.90	1.07	0.15	23.68
SVR	228.76	1.83	1.97	0.04	2.73	1.15	0.37	1.33	0.57	0.73	0.15	23.67
ensemble	199.48	1.63	1.63	0.03	2.03	0.74	0.26	0.88	0.37	0.60	0.13	21.18

Overall, the LIBS–ML framework achieved excellent agreement with laboratory reference values for most targets, with *R* ^2^ ≥ 0.94 for 11 of the 12 outputs using the best performing model, and consistently low absolute errors (RMSE). The consistently strong performance of the weighted ensemble highlights the benefit of target specific model weighing, particularly for heterogeneous waste derived feedstocks where different regressors may capture different nonlinearities and matrix dependent behaviors.

In the present results, Inner RMSECV and external test RMSE are of comparable magnitude across targets, indicating that the optimized configurations (including the weighted ensemble) transfer well from the tuning stage to unseen data. Differences between Inner RMSECV and external RMSE are expected because the inner CV evaluates multiple smaller training/validation partitions drawn from the development subset, while the external test set can exhibit slightly different composition and variability. Importantly, the absence of a systematic pattern where Inner RMSECV is not consistently much lower than the external RMSE suggests there is not overfitting during tuning and supports the conclusion that the reported external performance reflects genuine generalization rather than overfitting to the optimization procedure.

Across nearly all targets, the MLP (ANN) delivered outstanding performance, indicating that nonlinear mapping between selected LIBS spectral features and the laboratory targets is captured effectively by artificial neural network. The weighted ensemble method matched or slightly improved the best base model performance for most properties (e.g., HHV MAF *R* ^2^ = 0.98 with the lowest RMSE of 205.74; Ash, db (dry basis) *R* ^2^ = 0.99 with RMSE 1.25%), demonstrating the expected variance reduction benefit of model averaging when base learners provide complementary error patterns. Practically, this is important for online gasifier operation because the ensemble offers a more stable “default” predictor across many outputs, minimizing sensitivity to sample heterogeneity and spectral variability.

For the core operational variables, the models were highly predictive: HHV MAF, ash, and fixed carbon all reached *R* ^2^ ≈ 0.98–0.99 for the MLP and ensemble, with low RRMSE values (e.g., HHV MAF RRMSE 1.88% for the ensemble). Sulfur also showed strong *R* ^2^ (0.97) and low RMSE (0.04%) but comparatively high RRMSE (∼15%). This behavior is expected when the mean sulfur content (0.025%) is very small. Even small absolute deviations (that are near the analytical noise floor of lab reference methods) translate into inflated relative error metrics. Thus, sulfur’s RRMSE is primarily a scale effect, rather than evidence of poor trend-capture (as reflected by the high *R* ^2^).

The oxide predictions were uniformly strong (many with *R* ^2^ ≈ 0.98–1.00), confirming that the selected emission line feature sets retain sufficient chemical sensitivity for quantitative inference of ash forming species. Notably, the MLP showed exceptional performance for CaO, MgO, and K_2_O (e.g., CaO *R* ^2^ = 1.00, MgO *R* ^2^ = 0.99, K_2_O *R* ^2^ = 0.99), suggesting that these targets benefit from the ANN’s ability to learn nonlinear relationships and cross element spectral interactions (matrix effects) that are difficult to represent with simpler kernels or tree splits.

In contrast, Na_2_O behaved differently from the other oxides, with RF providing the best *R* ^2^ (0.95) compared with MLP/ensemble (0.94) and SVR (0.93). This indicates that sodium inference may be more dependent on piecewise relationships (e.g., regime like behavior due to self-absorption tendencies, local matrix dependence, or class specific spectral response), for which tree ensembles can be particularly effective. Importantly, RMSE for Na_2_O remained low and similar across models (∼0.09–0.10), so the practical difference is mainly in explained variance (*R* ^2^) rather than large absolute error.

Initial deformation temperature exhibited the lowest *R* ^2^ across all targets (best *R* ^2^ = 0.79 for MLP/ensemble), yet its relative error remained small (ensemble RRMSE 1.66%, RMSE 41.6 °F). When the true temperature range is relatively constrained (or clustered) compared with the inherent uncertainty of the laboratory measurement, *R* ^2^ can decrease even if absolute errors remain modest. Moreover, for high temperature measurements, laboratory accuracy typically degrades in absolute terms (e.g., a small percentage uncertainty corresponds to tens of degrees at elevated temperatures), which further compresses the explainable variance. Therefore, the reduced *R* ^2^ for initial deformation temperature is physically and metrologically reasonable, and the achieved RMSE still supports online decision making where temperature is used as a control-relevant indicator rather than a strictly metrological end point.

In Figure 4, across HHV MAF (BTU/lbm), ash, fixed carbon, and the major ash-forming oxides, initial deformation temperature the predicted values cluster closely around the 1:1 line, with most points lying within the ±10% band, confirming strong generalization to unseen samples spanning biomass, waste coal, and blended feedstocks as shown in Figure 4. The ensemble and MLP models exhibit the tightest alignment with the parity line, consistent with their highest *R* ^2^ and lowest RMSE/RRMSE for most targets. Sulfur shows visibly higher relative scatter, particularly at low concentrations, which is expected because small absolute deviations translate into larger percentage errors when the sulfur content is near the analytical noise floor. Initial deformation temperature displays comparatively lower *R* ^2^ despite moderate absolute error, attributable to the limited/clustered temperature range and increasing measurement uncertainty at elevated temperatures. Overall, the parity plots corroborate the quantitative metrics and demonstrate that the ensemble (and especially the MLP/ANN component) provides reliable online predictions for key gasifier-relevant feedstock properties. Parity plots for other parameters are shown in the appendix (Supporting Information) section.

**Figure 4:**
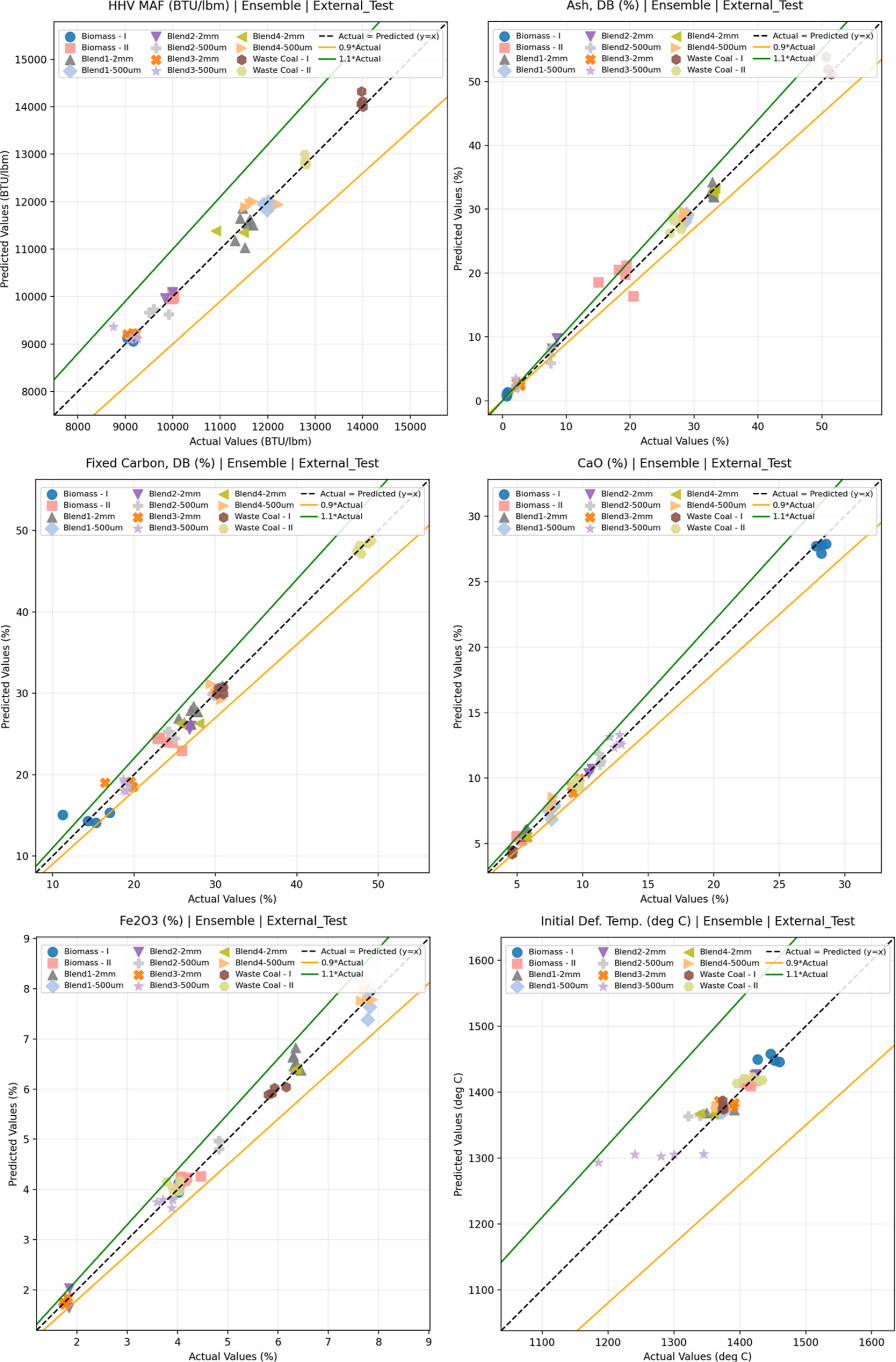
Parity plots for HHV MAF (BTU/lbm), Ash Analysis, Fixed Carbon, CaO, Fe_2_O_3_ and Initial Deformation Temp. Prediction.

Collectively, these results demonstrate that an online LIBS–ML regression pipeline can deliver near lab-quality predictions for HHV, ash content, fixed carbon, and key slagging/fouling-related oxides in real time. The ensemble predictor is recommended as the primary deployment model due to its broad robustness across targets, while the MLP remains the strongest single model, especially for CaO, MgO, and K_2_O, where nonlinear spectral–composition coupling is prominent. For Na, deploying RF as a specialized submodel (or increasing its ensemble weight only for Na_2_O) is justified by its superior variance explanation. Finally, initial deformation temperature prediction should be interpreted with attention to measurement uncertainty and temperature range effects. Despite lower *R* ^2^, the error magnitude is consistent with high-temperature laboratory limitations and remains suitable for process monitoring and feed-forward adjustments.

### Sensitivity Analysis Based on Training Testing Data Set

The sensitivity analysis framework was developed to evaluate the stability and robustness of the machine learning ensemble method when subjected to variations in external test set size. This procedure is vital for obtaining an accurate estimate of the generalization error (or out-of-sample error), which indicates how reliably the model will perform on data instances it has never encountered during the training phase.

To evaluate the robustness of the proposed LIBS-based regression framework, a sensitivity analysis was conducted by systematically varying the hold-out test fraction. For each target property (HHV, ash, fixed carbon, sulfur, major ash-forming oxides, alkali oxides, and initial deformation temperature), a fixed set of target-specific predictor variables was used to train the previously optimized weighted ensemble regressor. The ensemble combined the three constituent learners (ANN/MLP, RF, and SVR) using target-dependent weights and tuned model settings obtained from earlier optimization, ensuring that the sensitivity study assessed performance stability rather than reoptimizing the model each time.

For each target, the sensitivity study used a series of independent train and test partitions at multiple test size levels (from 5% up to 45% in 0.05% increments, while maintaining, as much as possible, a similar distribution of feedstock classes in both subsets to avoid bias from unbalanced splitting. The test-set fractions from 5% to 45% were selected to evaluate model sensitivity across a broad range of practically relevant hold-out sizes. Small test fractions preserve more samples for training but can yield unstable external-test metrics because the evaluation is based on few observations, whereas larger test fractions provide a more representative and statistically stable test set but reduce the amount of data available for model fitting. The chosen range therefore captures both extremes of this trade-off, while the 5% step size offers enough granularity to identify performance trends without overcomplicating the analysis. At each test size, the data set was randomly split ten times using different random seeds and splits were generated using class-stratified train/test partitioning.

The optimized ensemble model (with fixed target-specific features and previously determined weights (hyperparameters) was trained on the corresponding training subset and evaluated on the hold-out subset. Performance was quantified for every split using RMSE, *R* ^2^, and RRMSE (%). The resulting distributions at each test size were then summarized by reporting the mean ± standard deviation of each metric, providing an estimate of both the expected predictive accuracy and its variability due to sampling uncertainty. Across all targets, the 15% test split produced the weakest mean performance and/or the largest variability (higher RMSE/RRMSE and lower *R* ^2^), while performance generally stabilized for test sizes ≥20%, consistent with more reliable generalization estimates at moderate hold-out fractions.

Table 5 summarizes the sensitivity of external set performance to the hold-out (external test) fraction (5%–45%) by reporting the minimum, maximum, and average RMSE, *R* ^2^, and RRMSE (%) across all split conditions. Overall, the models show stable generalization: HHV MAF remains consistently accurate (RMSE 170.77–219.32, *R* ^2^ 0.98–0.99, RRMSE 1.57–2.02%), and the proximate properties (Ash, Fixed Carbon, Sulfur) maintain high predictive strength (e.g., Ash RMSE 1.06–1.51, *R* ^2^ 0.99; Fixed Carbon *R* ^2^ 0.94–0.97). For the alkali content, performance is generally strong (average *R* ^2^ 0.97–0.98 for most oxides), while SiO_2_ (*R* ^2^ 0.91–0.98) and Na_2_O (*R* ^2^ 0.88–0.93) exhibit the greatest sensitivity to test size. Initial deformation temperature shows the lowest *R* ^2^ and widest spread (0.65–0.84, avg 0.75) with RMSE 30.49–45.21 °F (avg 38.34 °F), indicating weaker explainability. However, it simultaneously achieves the lowest relative error among all targets (RRMSE 1.21–1.79%, avg 1.52%), reflecting a small percentage deviation relative to the magnitude of the temperature response. Additional sensitivity analysis graphs are available in the appendix (Supporting Information) section.

**Table 5:** Sensitivity Analysis Summary Results

	RMSE			*R* ^2^			RRMSE (%)		
target	max	min	average	max	min	average	max	min	average
HHV MAF (BTU/lbm)	219.32	170.77	192.23	0.99	0.98	0.98	2.02	1.57	1.77
Ash, DB (%)	1.51	1.06	1.35	0.99	0.99	0.99	7.45	5.21	6.66
fixed carbon, DB (%)	1.92	1.44	1.59	0.97	0.94	0.96	7.21	5.40	5.96
sulfur, DB (%)	0.04	0.03	0.04	0.98	0.97	0.97	15.99	13.82	14.89
SiO_2_ (%)	3.09	1.57	2.02	0.98	0.91	0.96	6.42	3.26	4.20
Al_2_O_3_ (%)	1.19	0.56	0.71	0.99	0.95	0.98	5.94	2.77	3.51
Fe_2_O_3_ (%)	0.41	0.17	0.24	0.99	0.95	0.98	8.47	3.57	4.99
CaO (%)	1.45	0.48	0.80	0.99	0.93	0.98	14.66	4.84	8.12
MgO (%)	0.61	0.21	0.40	0.99	0.95	0.97	18.94	6.50	12.51
K_2_O (%)	0.82	0.52	0.62	0.98	0.96	0.98	17.12	10.94	12.93
Na_2_O (%)	0.14	0.11	0.12	0.93	0.88	0.91	15.78	12.68	13.53
IDT (°C)	45.21	30.49	38.34	0.84	0.65	0.75	1.79	1.21	1.52

Furthermore, the analysis aims to mitigate sampling bias and data snooping bias, risks that occur when a test set is too small to accurately represent the data distribution, leading to artificially optimiztic or imprecise results.^37^ Ultimately, this sensitivity analysis serves as a validation of the ensemble method’s discriminatory power and its ability to maintain high confidence levels across diverse data scenarios.

## Conclusion

This study demonstrates that online feedstock characterization for heterogeneous waste-derived gasifier fuels can be achieved by coupling conveyor-based LIBS with leakage-safe machine learning regression. A controlled sample matrix containing waste coal, biomasses, and engineered blends (evaluated across two particle size conditions) was designed to represent composition and physical variability relevant to gasifier operation. To improve robustness under dynamic, belt-fed measurement conditions, each spectral data point was formed by averaging 800 consecutive laser pulses, and repeatability was verified using an RSD-based screening criterion, yielding a stable data set of 240 representative spectra (20 observations per group).

A supervised regression framework was implemented using three complementary model families (MLP/ANN, RF, and SVR) to map LIBS-derived emission line features to gasifier-relevant targets. Model complexity and ensemble construction were handled with a nested 5-fold cross-validation strategy, where Bayesian optimization tuned hyperparameters in the inner loop, and a weighted ensemble with optimized settings was constructed. Final performance was reported using both cross-validated predictions and an independent external hold-out set, supported by parity plots with ±10% tolerance bands to assess practical agreement and bias.

Across most outputs, the LIBS–ML approach achieved near-laboratory predictive fidelity: 11 of 12 targets reached *R* ^2^ ≥ 0.94 for the best performing model, with the weighted ensemble providing the most reliable “default” predictor across properties (e.g., HHV MAF *R* ^2^ = 0.98 with RMSE ≈205.7 BTU/lbm; ash DB *R* ^2^ = 0.99 with RMSE ≈1.25%). Sodium behavior was comparatively model-dependent, with RF providing the highest explained variance for Na_2_O (*R* ^2^ ≈ 0.95), suggesting more regime-like response patterns for this species. Initial deformation temperature showed the lowest *R* ^2^ (best ≈0.79) yet retained modest absolute error (RMSE ≈41.6°F; RRMSE ≈1.66%), consistent with limited inherent uncertainty of the laboratory measurement at elevated temperatures. Finally, a repeated split sensitivity analysis indicated that performance generally stabilizes at moderate hold-out fractions (≥0.20), supporting the robustness of the proposed online regression framework for deployment-relevant generalization estimates.

In future deployments, LIBS ML predictions can function as a real feed forward “fuel quality signal” that integrates into the plant control system upstream of the reaction zone. As each belt segment is scanned, instantaneous measurements of HHV, ash content, and ash/oxide composition translate into actionable feed quality indices including expected oxygen demand, slagging risk, and alkali risk. These indices can then be sent to the gasifier Distributed Control System/Programmable Logic Controller at the same rate as the conveyor measurement. This allows the operator or supervisory logic to adjust operating targets in advance, so the gasifier is ready for the next fuel parcel and helps maintain a stable temperature profile, protects syngas quality (H_2_/CO and heating value), and reduces unexpected issues when different waste blends arrive. The forward signal also informs routing and blending strategies, such as modulating feed rate, introducing buffer fuel, or rerouting out of specification material thereby preventing high ash or high alkali excursions that could trigger slagging or fouling. This framework aligns with DOE/NETL priorities emphasizing how real time feedstock characterization enables feed forward gasifier control for variable blended fuels^41^

Moving forward, expanding the training library to broader waste streams, and operating conditions, improving temporal resolution (while preserving repeatability), and incorporating complementary sensing (e.g., LIBS–Raman data fusion) would further strengthen resilience to matrix effects and enable tighter integration with feed forward gasifier control strategies.

## Supplementary Material



## Notes

The Supporting Information contains additional parity plots for the predicted alkali oxide contents (SiO_2_, Al_2_O_3_, TiO_2_, Fe_2_O_3_, CaO, MgO, K_2_O, Na_2_O) and test-size sensitivity analysis graphs for all 12 target properties (HHV MAF, ash, fixed carbon, sulfur, major ash-forming oxides, and initial deformation temperature). These Supporting Information figures provide extended validation of the LIBS–ML regression framework across varying hold-out fractions and prediction targets (PDF)
