# Supplementary material for: Online LIBS–ML Framework for Dynamic Characterization of Heterogeneous Waste-Derived Gasification Feedstocks
Source: ACS Omega. 2026 May 20;11(21):31729–43. doi: 10.1021/acsomega.6c03040 (PMC13234666; doi:10.1021/acsomega.6c03040)
Supplement: Supplementary file 1 [file ao6c03040_si_001.pdf]

Supporting Information

Online LIBS–ML Framework for Dynamic Characterization of Heterogeneous Waste-Derived Gasification Feedstocks

Ozan Karadut,[1,2] Javidan Aliyev,[1,3] Carlos E. Romero,[1,2] Zheng Yao,[1,2] Robert De Saro,[4] Joseph Craparo[4]

[1]Energy Research Center, Lehigh University, Bethlehem, PA 18015, USA
[2]Department of Mechanical Engineering and Mechanics, Lehigh University, Bethlehem, PA 18015, USA
[3]Department of Chemical and Biomolecular Engineering, Lehigh University, Bethlehem, PA 18015, USA
[4]Energy Research Company, 400 Leland Ave, Plainfield, NJ 07062, USA

Corresponding Author: sok522@lehigh.edu

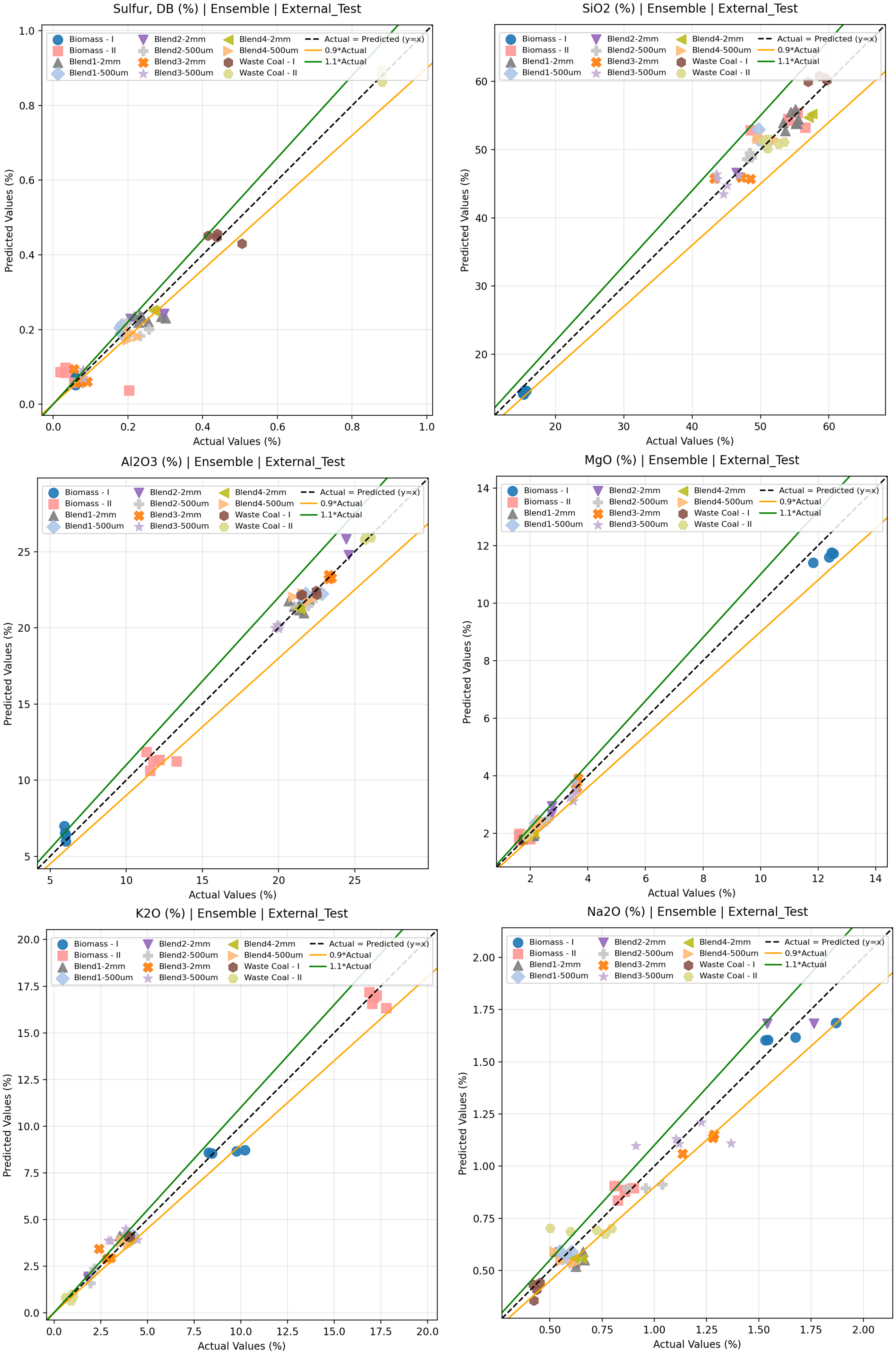

Figure S1: Parity plots for alkali contents.

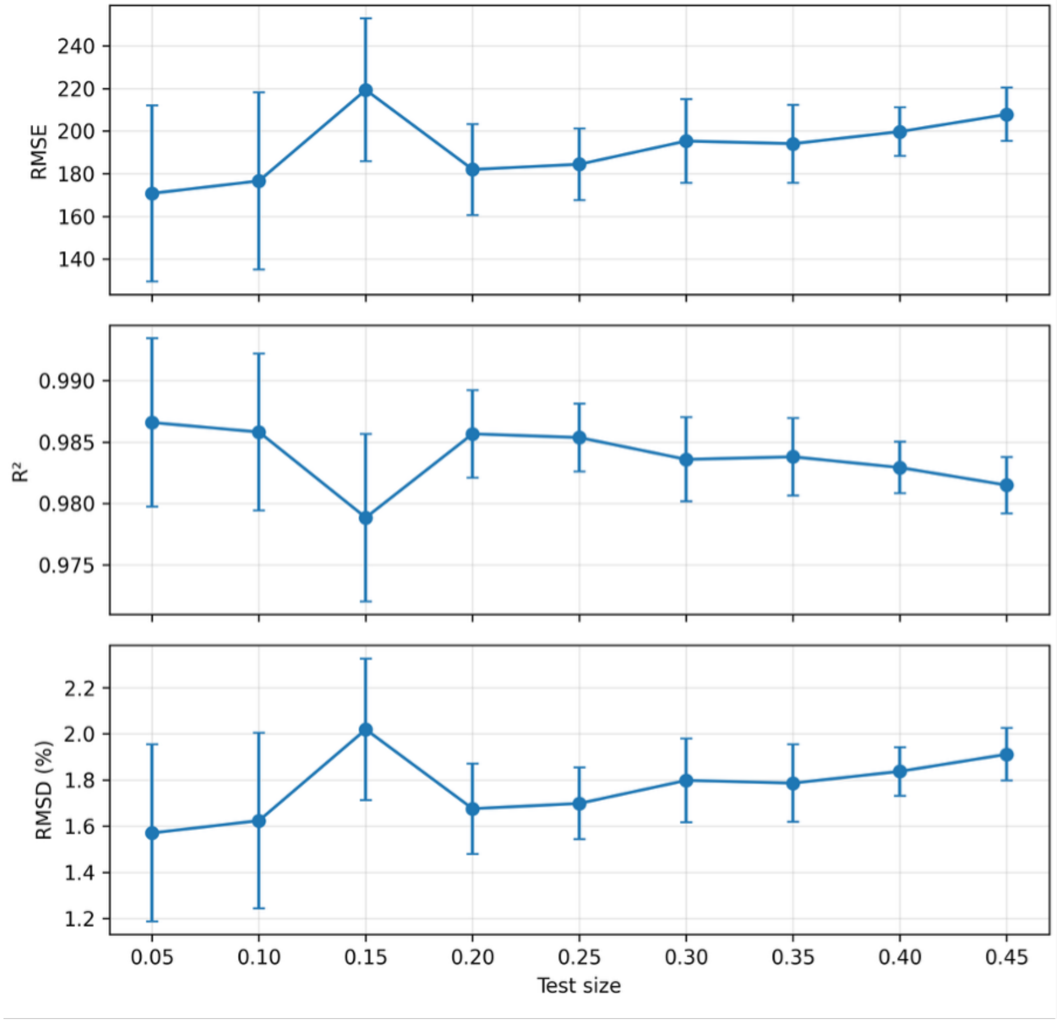

Figure S2: HHV MAF Test-size sensitivity analysis

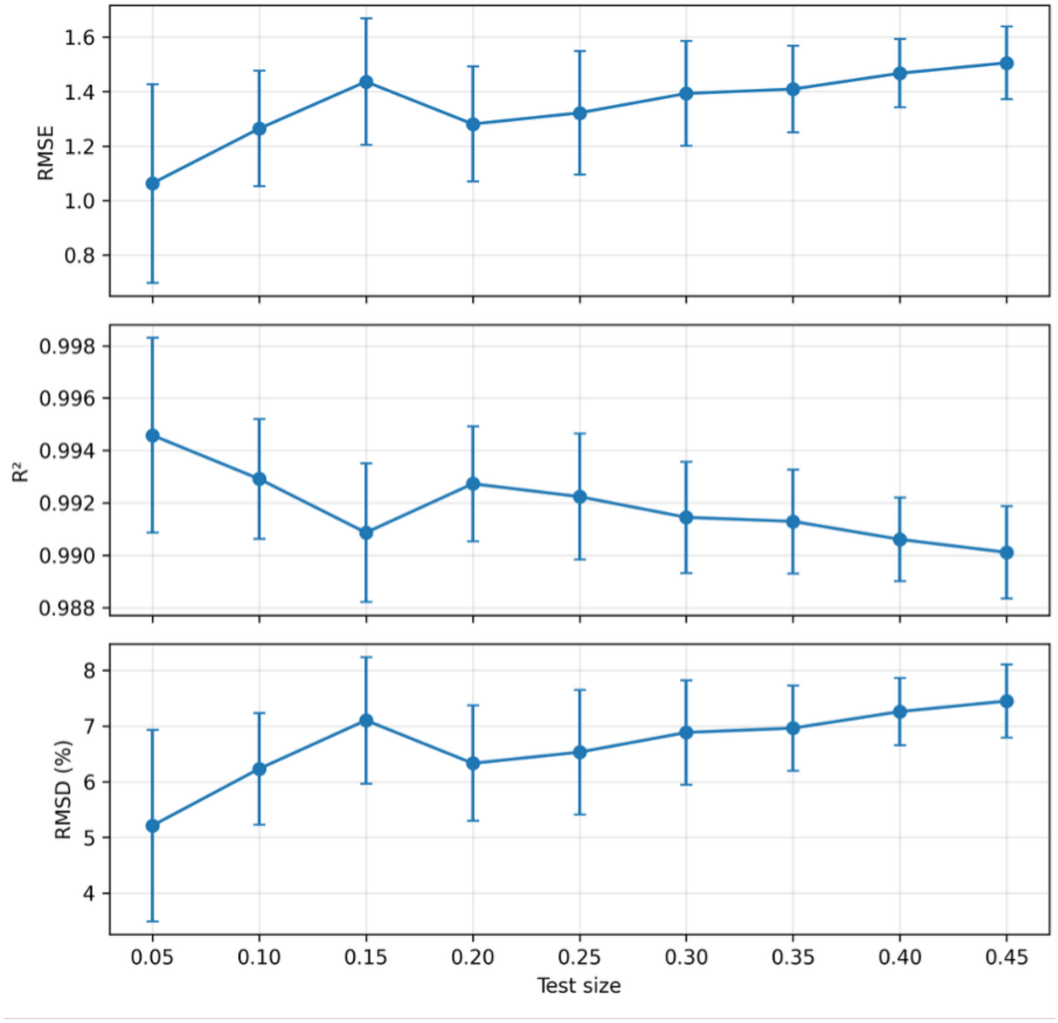

Figure S3: Ash, db Test-size sensitivity analysis

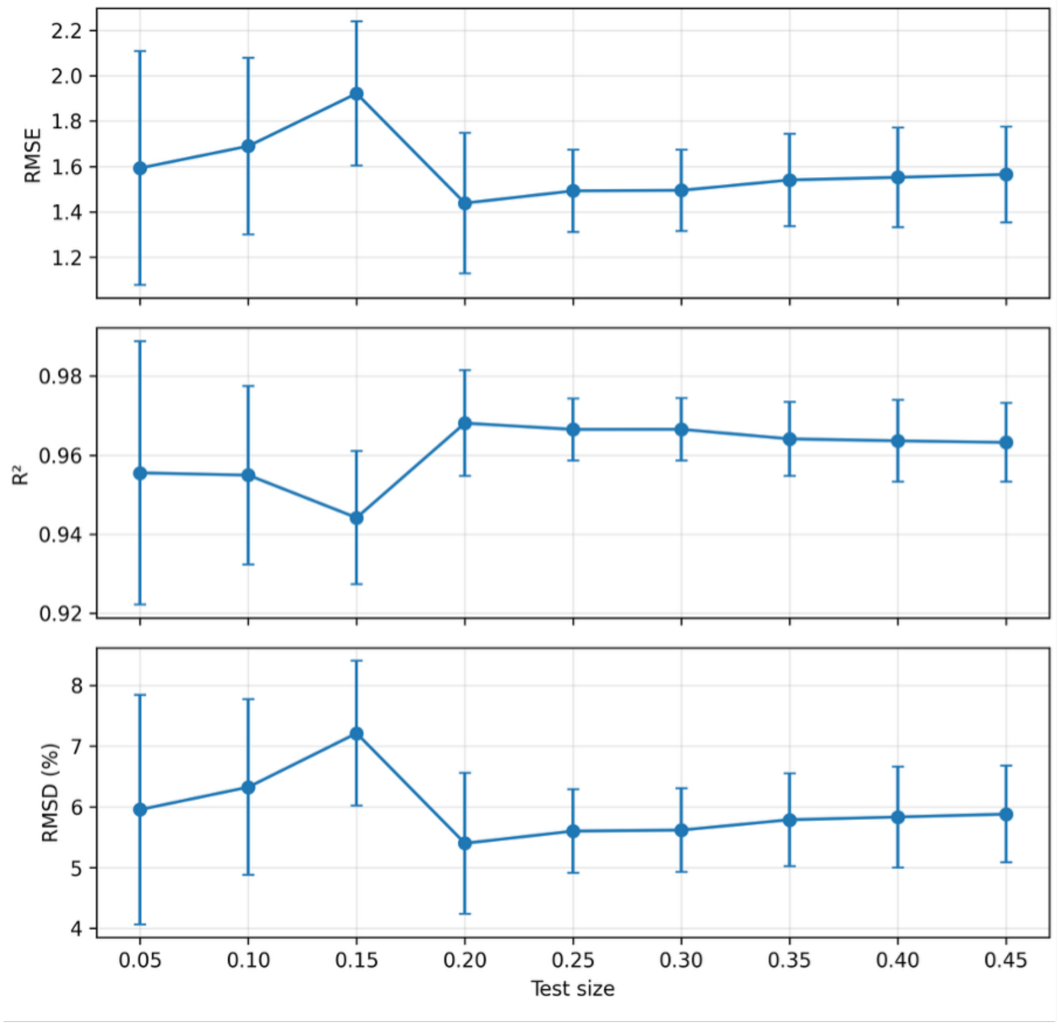

Figure S4: Fixed Carbon, DB Test-size sensitivity analysis

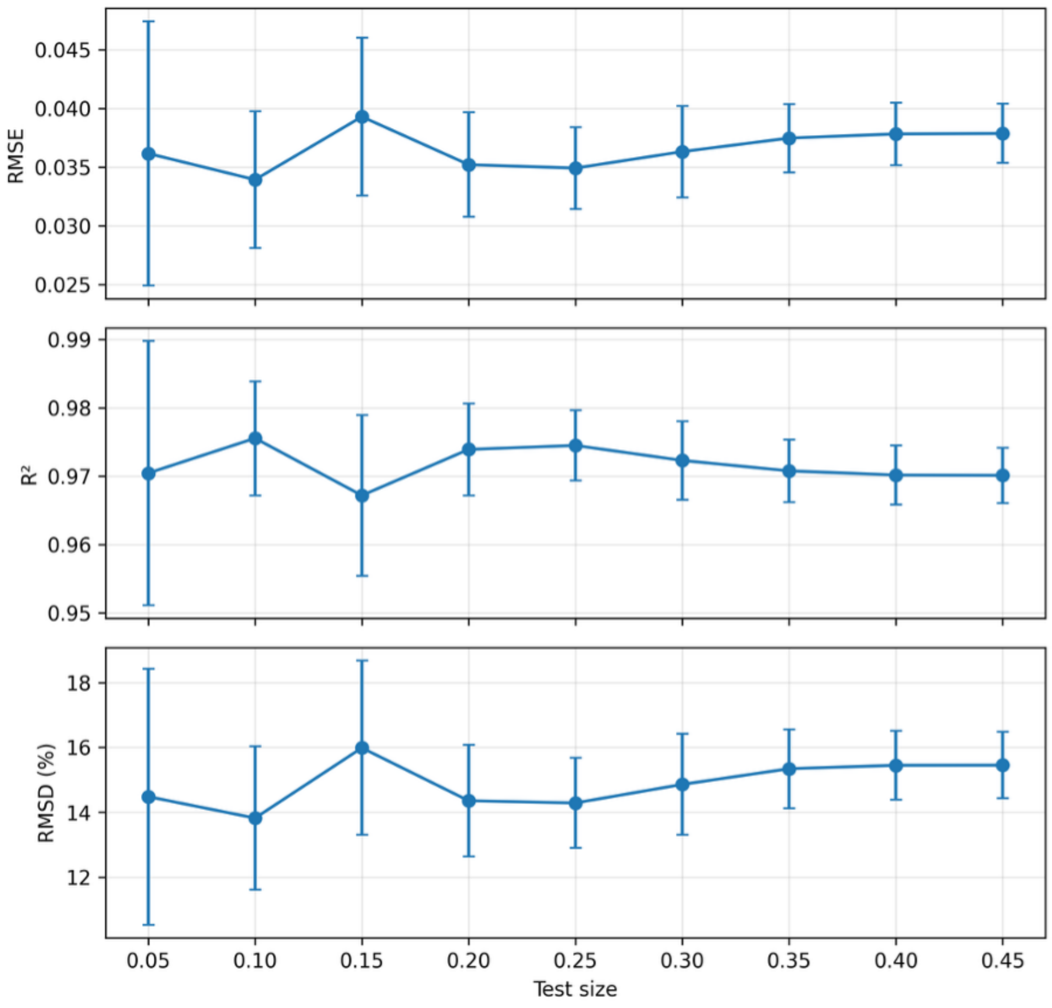

Figure S5: Sulfur, DB (%) Test-size sensitivity analysis

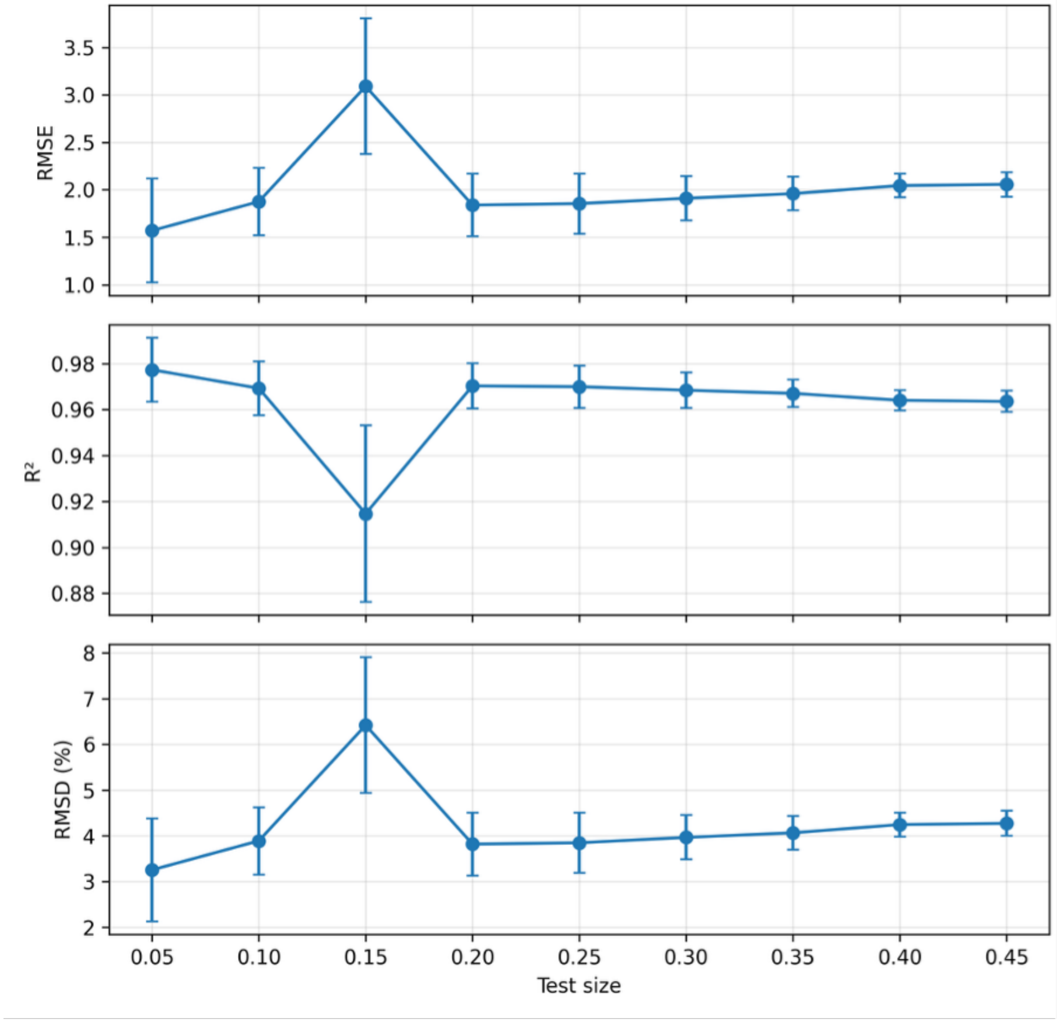

Figure S6: SiO_2 Test-size sensitivity analysis

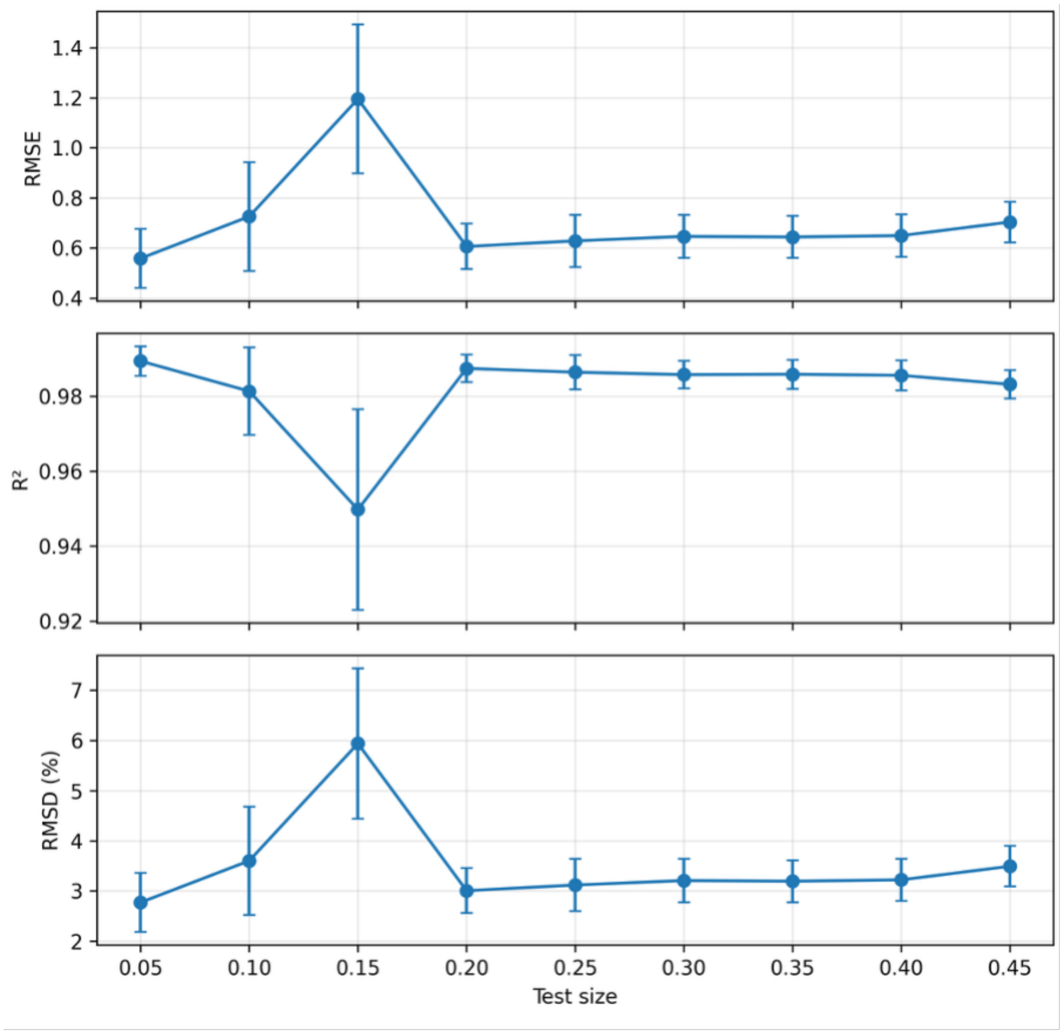

Figure S7: Al_2O_3 Test-size sensitivity analysis

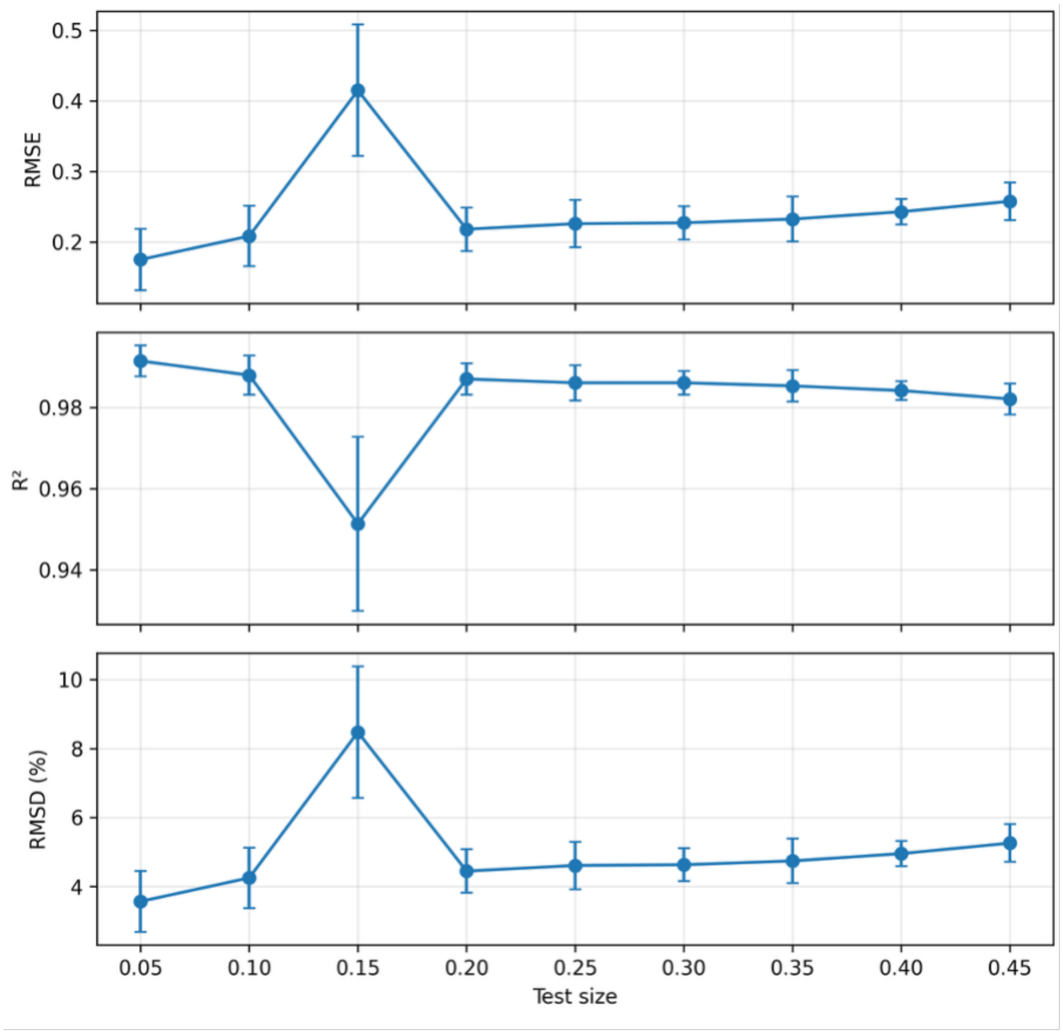

Figure S8: Fe_2O_3 Test-size sensitivity analysis

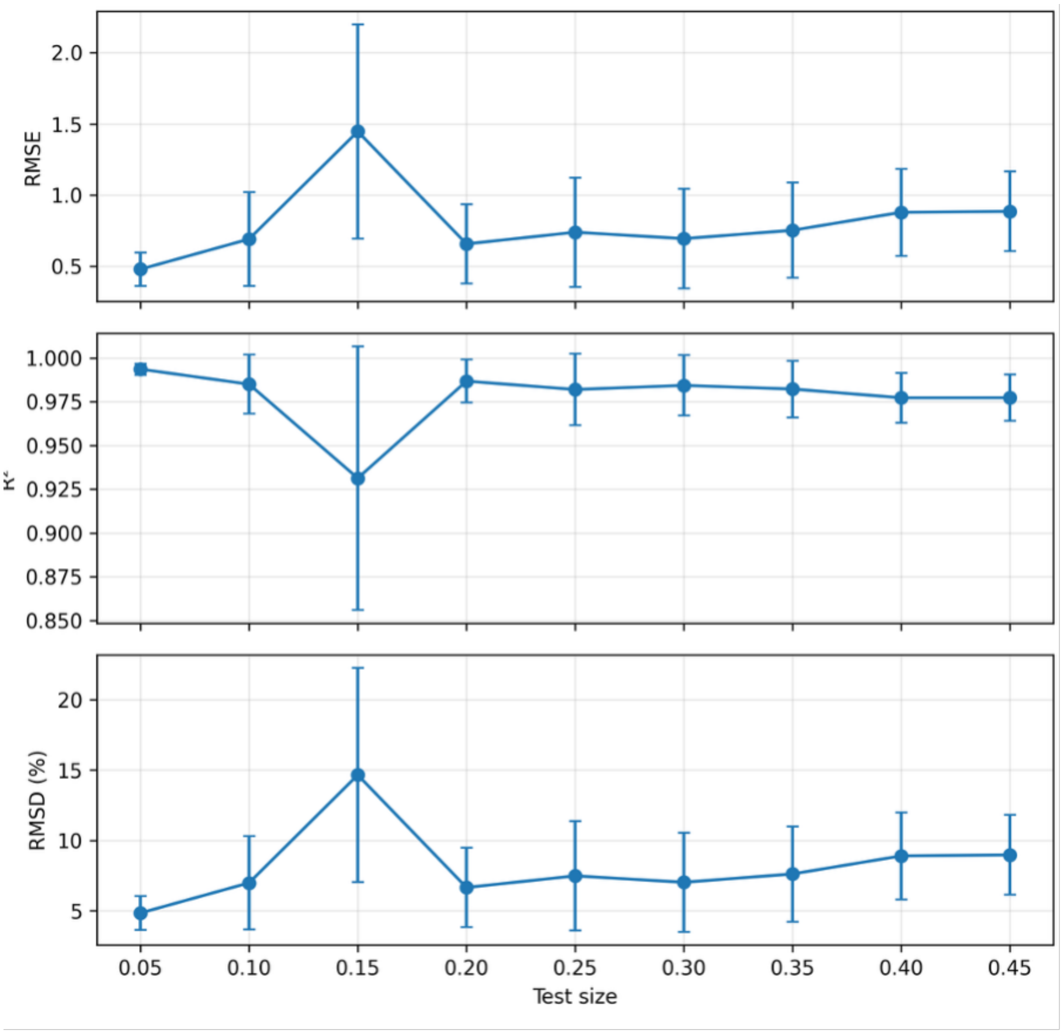

Figure S9: CaO Test-size sensitivity analysis

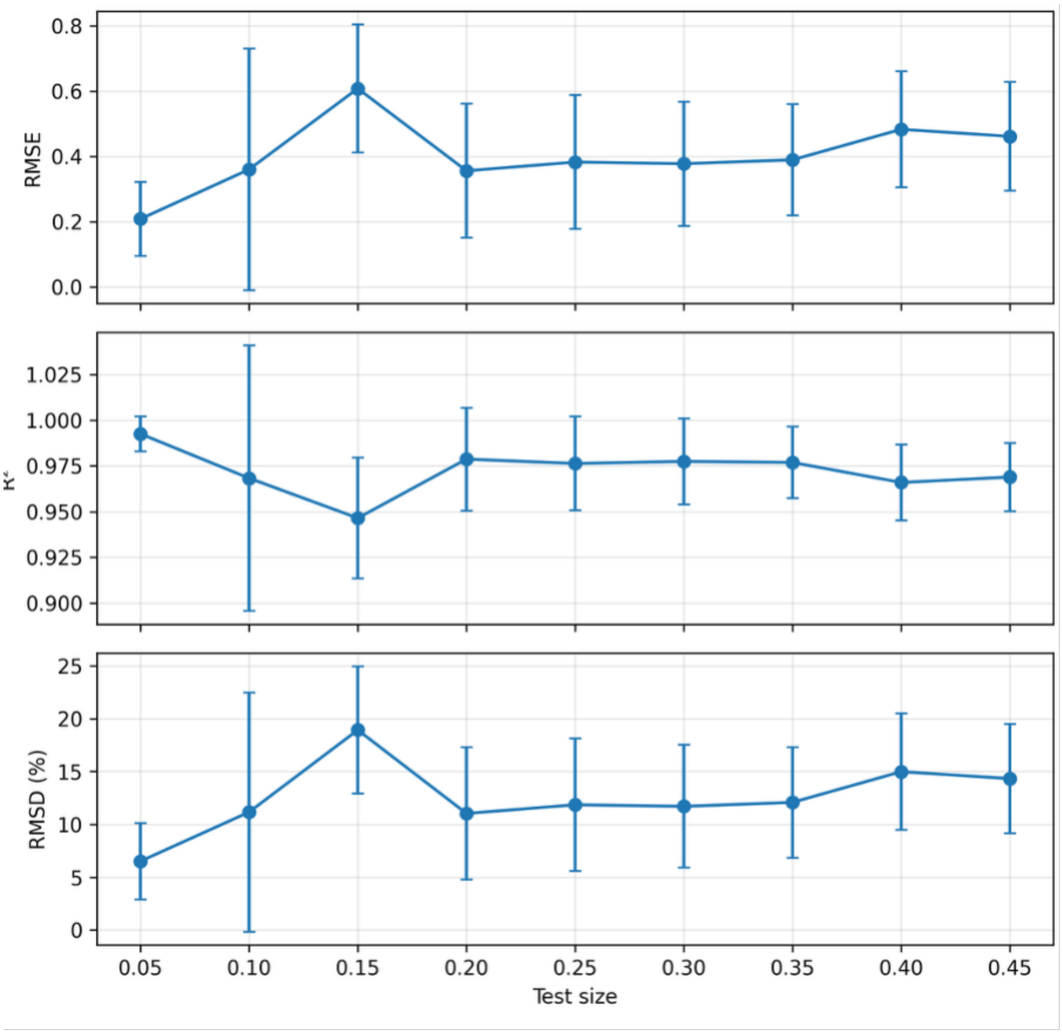

Figure S10: MgO Test-size sensitivity analysis

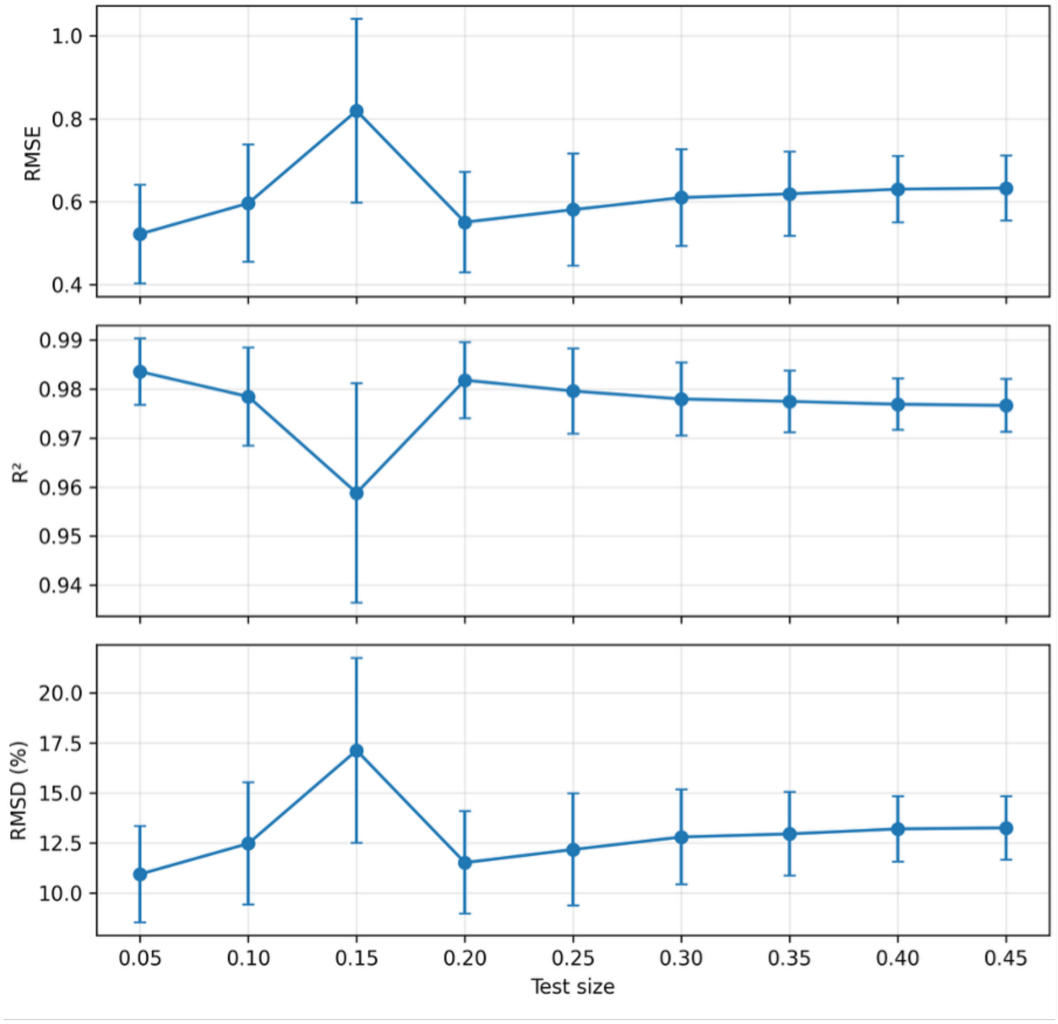

Figure S11: K_2O Test-size sensitivity analysis

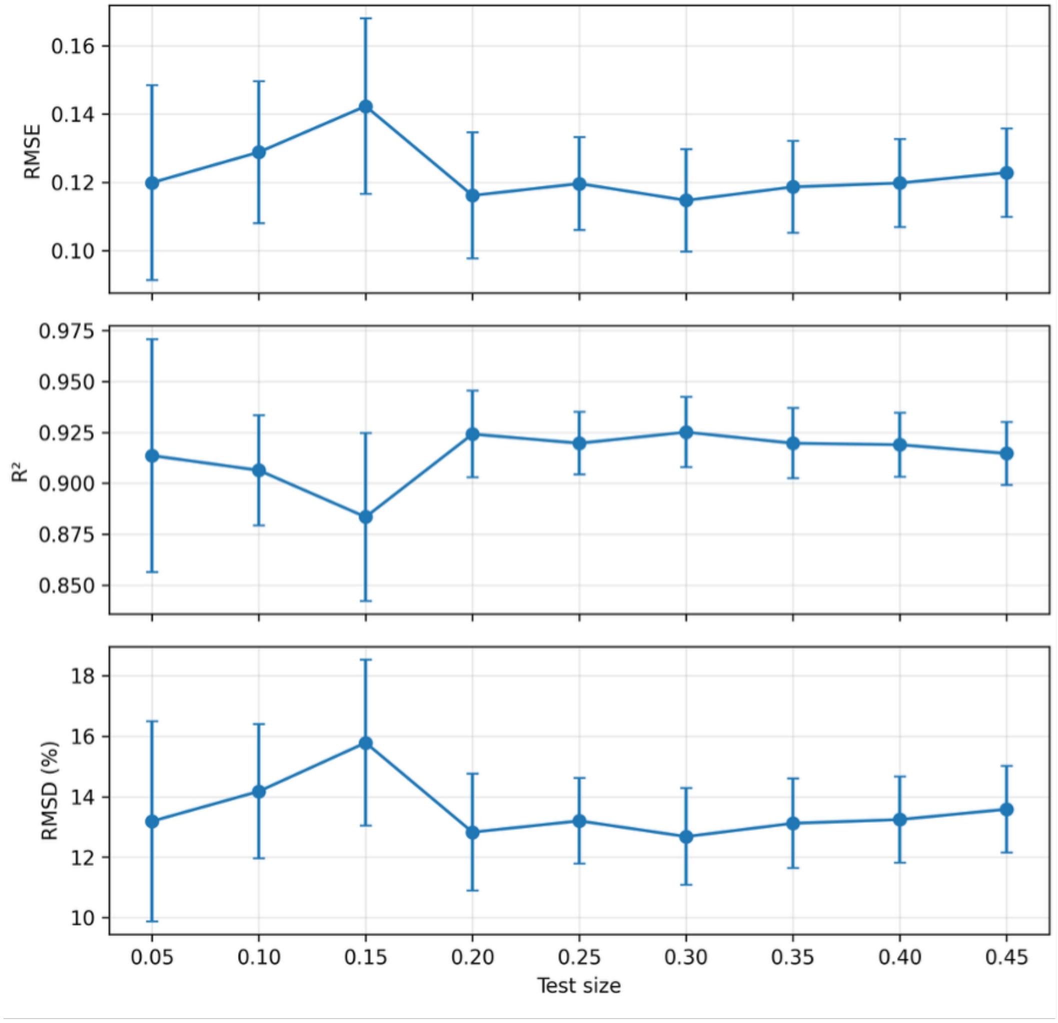

Figure S12: Na_2O Test-size sensitivity analysis

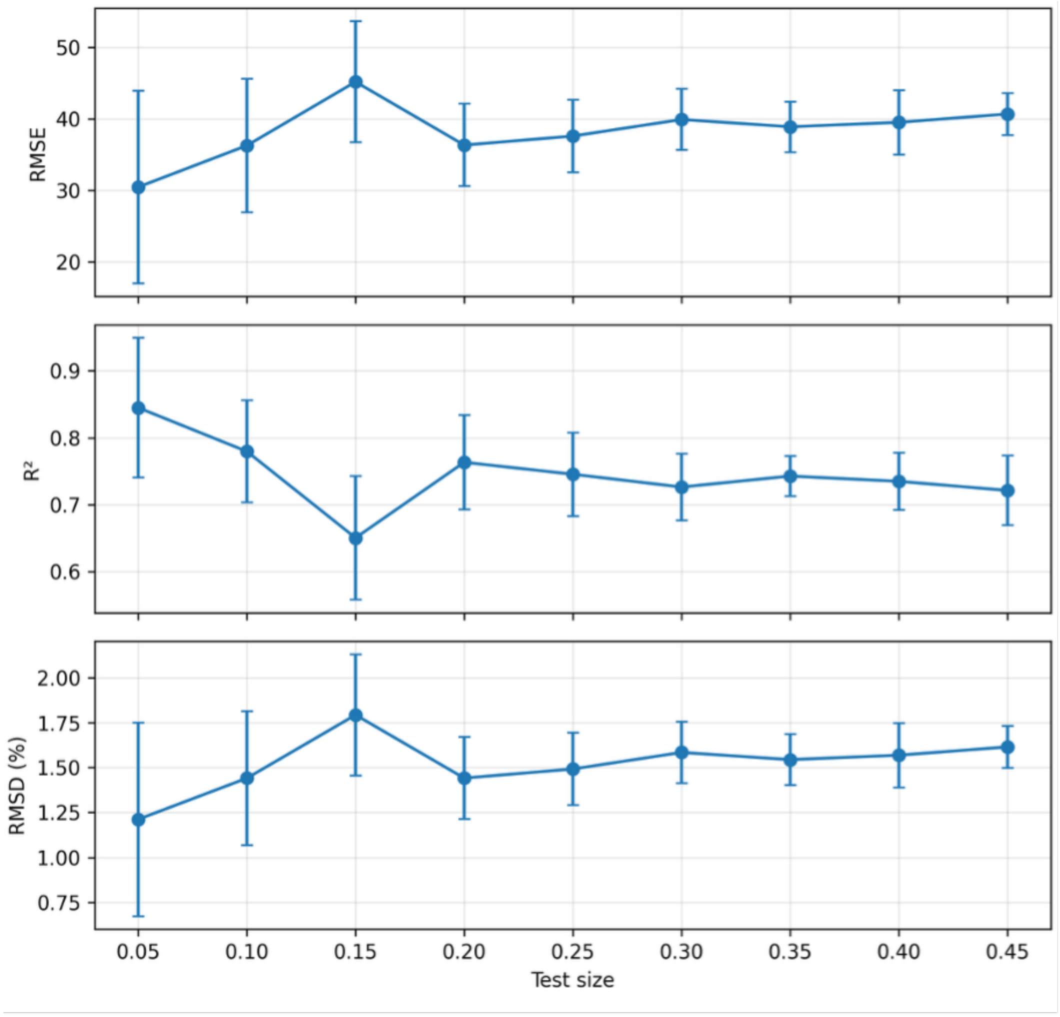

Figure S13: IDT Test-size sensitivity analysis
